# Redox integration of signaling and metabolism in a head and neck cancer model of radiation resistance using COSM^RO^

**DOI:** 10.3389/fonc.2022.946320

**Published:** 2023-01-04

**Authors:** Zhiwei Ji, Jade Moore, Nelmi O. Devarie-Baez, Joshua Lewis, Hanzhi Wu, Kirtikar Shukla, Elsa I. Silva Lopez, Victor Vitvitsky, Chia-Chi Chuang Key, Mercedes Porosnicu, Melissa L. Kemp, Ruma Banerjee, John S. Parks, Allen W. Tsang, Xiaobo Zhou, Cristina M. Furdui

**Affiliations:** ^1^ Division of Radiologic Sciences – Center for Bioinformatics and Systems Biology, Wake Forest School of Medicine, Winston-Salem, NC, United States; ^2^ Department of Internal Medicine, Section on Molecular Medicine, Wake Forest School of Medicine, Winston-Salem, NC, United States; ^3^ The Parker H. Petit Institute of Bioengineering and Bioscience, Georgia Institute of Technology, Atlanta, GA, United States; ^4^ The Wallace H. Coulter Department of Biomedical Engineering, Georgia Institute of Technology, Emory School of Medicine, Atlanta, GA, United States; ^5^ Department of Biological Chemistry, University of Michigan Medical School, Ann Arbor, MI, United States; ^6^ Department of Internal Medicine, Section on Hematology and Oncology, Wake Forest School of Medicine, Winston-Salem, NC, United States

**Keywords:** redox metabolism, head and neck cancer, mixed integer programming, flux balance analysis, cholesterol metabolism, steroidogenesis, radiation response

## Abstract

Redox metabolism is increasingly investigated in cancer as driving regulator of tumor progression, response to therapies and long-term patients’ quality of life. Well-established cancer therapies, such as radiotherapy, either directly impact redox metabolism or have redox-dependent mechanisms of action defining their clinical efficacy. However, the ability to integrate redox information across signaling and metabolic networks to facilitate discovery and broader investigation of redox-regulated pathways in cancer remains a key unmet need limiting the advancement of new cancer therapies. To overcome this challenge, we developed a new constraint-based computational method (COSM^ro^) and applied it to a Head and Neck Squamous Cell Cancer (HNSCC) model of radiation resistance. This novel integrative approach identified enhanced capacity for H_2_S production in radiation resistant cells and extracted a key relationship between intracellular redox state and cholesterol metabolism; experimental validation of this relationship highlights the importance of redox state in cellular metabolism and response to radiation.

## Introduction

Ionizing radiation is widely used to treat cancer, and more than 50% of cancer patients receive either definitive or adjuvant radiation therapy (1). However, the therapeutic outcome of radiation is difficult to predict and is often undermined by the fraction of tumor cells that resist radiation damage. These tumor cells are generally characterized by increased expression of proteins with antioxidant functions, enabling suppression of basal and radiation-induced reactive oxygen species (ROS) and cellular damage (2). Thus, deciphering the mechanisms by which radiation resistant tumors reprogram redox metabolism to prevent and repair the damage induced by radiation is key to identifying targets that will sensitize tumors to radiation therapies.

**Figure 1:**
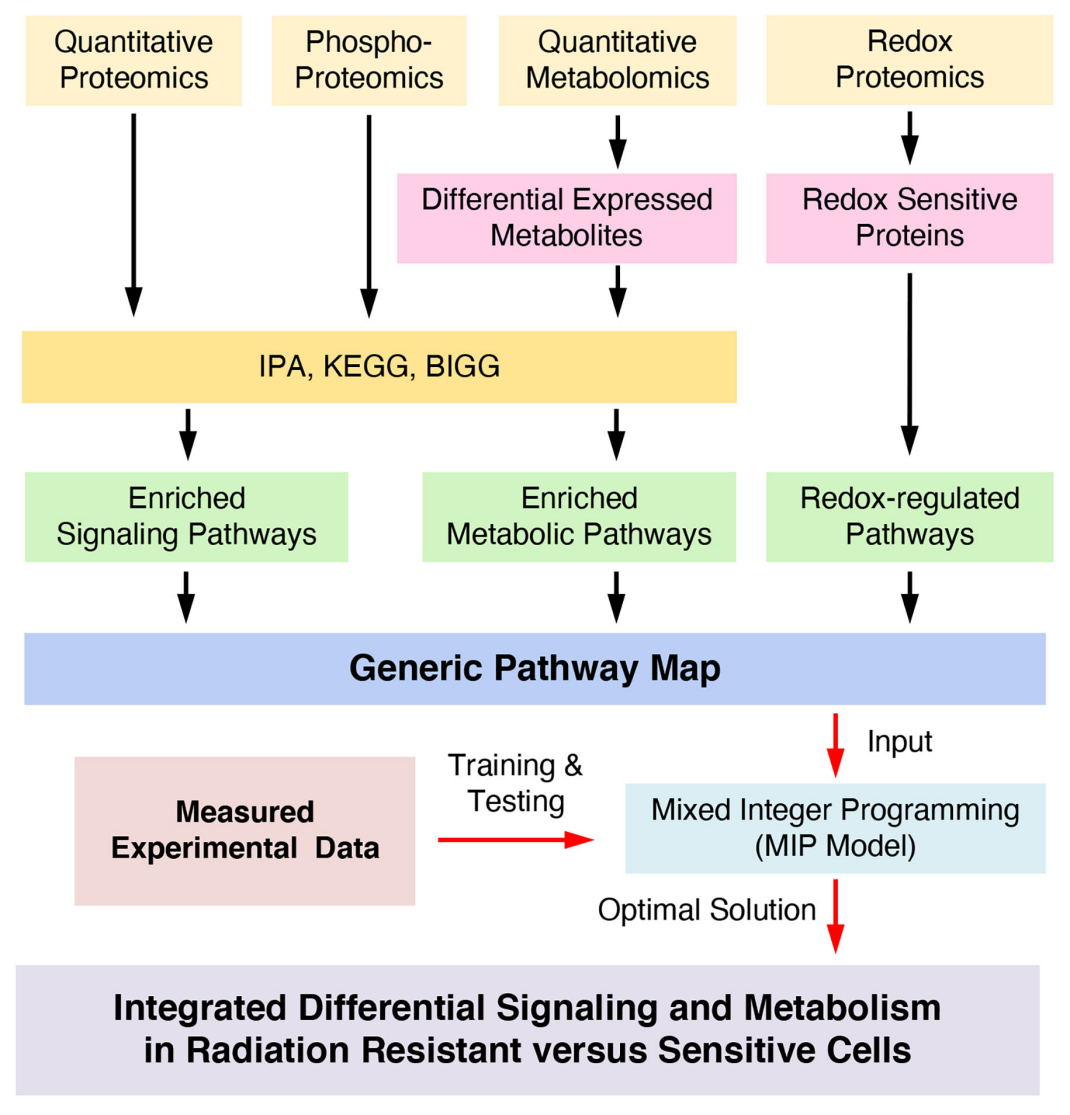
Mathematical workflow to integrate Redox effects on signaling and metabolism.

To discover new redox-integrated networks underlying the radiation resistance phenotype, we developed and present here a new constraint-based systemic modeling of integrated redox signaling and metabolic networks (COSM^ro^) method based on multi-omics and targeted data (**Figure 1**). As a case study, we focused on Head and Neck Squamous Cell Cancer (HNSCC), a highly heterogeneous disease frequently treated with radiation therapy as a key component of cancer management. We previously reported the development of a matched cell model of response to radiation in HNSCC (radiation sensitive SCC-61 cells and the radiation resistant derivative rSCC-61 cells). Comprehensive characterization of this system using systems biology approaches (3–6), identified common radiation resistance themes in rSCC-61 including the upregulation of DNA damage repair and antioxidant systems, aligned with the core mechanisms of radiation resistance reported across cancers (3).

The COSM^ro^ analysis of the SCC-61/rSCC-61 system produced data consistent with these findings, confirming the expected routing of glycolysis into the pentose phosphate pathway (PPP) needed to generate reducing equivalents in the form of NADPH and nucleotide building blocks for the repair of radiation-induced DNA damage. It also highlighted new processes such as H_2_S production and redox regulation of cholesterol metabolism defining the radiation response phenotype. Further investigation of mitochondrial 1-C metabolism focusing on the NADPH-producing methylene tetrahydrofolate dehydrogenase 2 (MTHFD2), has led to the discovery of MTHFD2-dependent steroidogenesis in rSCC-61 cells, a pathway previously thought to be present only in tumors of classical steroidogenic tissues (e.g., ovaries, testes). Steroidogenesis could be an important target for the treatment of non-adrenal cancers that may hijack this pathway to both lower the intracellular cholesterol and to produce metabolites such as cortisol, which support tumor growth and metastasis (7).

## Results

### COSM^ro^, a new constraint-based systemic mixed integer programming reveals key pathways associated with radiation resistance

#### Data input

In this study, we considered four types of data to infer a global redox-regulated cell-specific network. The proteomics data includes the total expression of 965 proteins, which are applied to screen enriched signaling pathways and metabolic enzymes (3). The metabolomics, redox proteomics and targeted protein phosphorylation data are described next.

#### Metabolomics analysis

The metabolic profiles of SCC-61 and rSCC-61 cells were compared to identify the metabolites that are significantly up- or down-regulated in rSCC-61. Data analysis using the ChemTOF software identified 195 peaks after applying the metabolite extraction, chemical derivatization and alignment procedures described in **Materials and Methods**. Principal component analysis was performed on all the samples where we found no obvious outliers for this dataset (2 components with R^2^X=0.713, Q^2 =^ 0.518) (**Figure S1A**). This was followed by supervised partial least square discriminant analysis (PLS-DA) to analyze the difference between SCC-61 and rSCC-61 cells. Three component PLS-DA models were obtained with R^2^X=0.783, R^2^Y=0.992 and Q^2 =^ 0.97 and the scores plot is shown in **Figure S1B**. The statistically different metabolites were selected according to the variable importance in the projection (VIP) (VIP>1) and *p* values from the student *t*-test (*p* < 0.05) (**Figure S1C**). The 38 identified differential metabolites from SCC-61 and rSCC-61 groups are listed in **Supplementary Dataset S1**. Additional metabolites were added from our previously published data (3).

#### Redox proteomics

Previous imaging analysis has shown decreased intracellular ROS in rSCC-61 compared with SCC-61 cells (3). To quantify the consequence on protein oxidation, the analysis of redox-regulated proteins in SCC-61 and rSCC-61 cells was performed using biotin-tagged BP1 probe (3) to label protein sulfenic acids (Cys-SOH) (**Figure S2A**) using the workflow shown in **Figure S2B**. The complete list of proteins quantified by this analysis is included in **Supplementary Dataset S2** and the plot of log2(Normalized Ratios) in **Figure S2C**. The Ingenuity Pathway Analysis (IPA, http://www.ingenuity.com) was used to determine the distribution of proteins subcellular locations (**Figure S2D**, upper chart), and to identify the molecular functions of redox-regulated proteins that are differentially enriched in the two cell lines (**Figure S2D**, lower chart). The relative abundance of five other proteins with antioxidant function were monitored by Western blot (**Figure S3A**).

#### Protein phosphorylation

The phosphorylation state of 12 proteins of interest was monitored by Western blot (**Figure S3B**). These proteins were selected based on their key function in connecting signaling and metabolism. The measured ratios of protein phosphorylation, the phosphorylation site monitored and the anticipated consequence on protein activity in SCC-61 and rSCC-61 cells are summarized in **Supplementary Dataset S3**.

### COSM^ro^ approach

To derive the redox-mediated network distinguishing the radiation sensitive and resistant cell lines, we applied joint optimization of signaling and metabolism *via* MIP. Linear programming is a novel approach for systemic modeling and network optimization. Some researchers developed integer linear programming (ILP) approaches to infer cell-specific signaling pathways and predict the drug treatment effects (8–11). MIP was also applied for metabolic flux balance analysis (12). However, there are few studies on how to optimize an integral network which includes both signaling and metabolic reactions. Besides, none of the methods that are currently available account for redox effects on the activity of proteins on a network-wide scale as described here.

#### Construction of a generic redox-regulated pathway map for SCC-61 and rSCC-61 cells

The information obtained from four datasets (proteomics (3), metabolomics, redox proteomics and protein phosphorylation described above and summarized in **Supplementary Datasets S1–S3** and **Figures S1–S3**) was used to build a redox-regulated generic network of integrated signaling and metabolic subnetworks (**Figure 1**, and **Figure S4**) as detailed in the **Supplementary Material**. The network was annotated with proteins connecting signaling and metabolic subnetworks (blue filled circles, **Figure S4**), and the effects of oxidation and phosphorylation on the activity of the proteins in the resulting network were annotated based on the literature and the experimental redox proteomics analysis of SCC-61 and rSCC-61 cells (overlapping proteins are shown in **Supplementary Table S1**). The redox-regulated signaling and metabolic proteins were connected to a node “H_2_O_2_” according to the information in **Supplementary Table S2**. Next, we represented signaling pathways as a Boolean network consisting of a set of nodes for signaling proteins (including some metabolic enzymes with dual function in signaling) and a set of directed edges (8, 11) representing activation ( → ) or inhibition ( −|) effects converging on downstream proteins. Examples of multiple activation, inhibition or mixed effects on downstream proteins are illustrated in **Figures S5A–C**. Similar to the signaling subnetwork, the metabolic subnetwork topology consists of a set of nodes and a set of edges. In this case, the nodes denote the metabolites in biochemical reactions and their measurements as ratios (fold change of expression) in rSCC-61 relative to SCC-61. The edges indicate reversible (duplex arrows) or irreversible (unidirectional arrows) reactions that are controlled by corresponding metabolic enzymes. The list of all metabolic subnetwork components including thermodynamic parameters for each metabolic reaction extracted from NIST Standard Database (13), BiGG (14), and Kyoto Encyclopedia of Genes and Genomes (15) is included in **Supplementary Dataset S4**.

The resulting topology of the generic integral network is shown in **Figure S4**. The upper portion of **Figure S4** is the signaling subnetwork, which consists of 54 signaling proteins (22 measured) and 10 metabolic enzymes (5 measured) connected through 87 regulatory signaling events (**Supplementary Dataset S5**). The lower portion of **Figure S4** is the metabolic sub-network which contains 74 metabolic reactions (37 metabolic enzymes measured) and 107 metabolites (38 metabolites measured).

#### Prediction of molecular features underlying the rSCC-61 phenotype using COSM^ro^

To infer the rSCC-61 specific pathway network based on the generic pathway map constructed, we minimized the differences between measurements and the simulated values, and the complexity of the network’s topology structure by developing a MIP-based approach to optimize such multi-objective functions (16, 17). MIP normalizes the states of the signaling proteins to binary variables “1” or “0”, which indicate upregulation or downregulation of protein activities in rSCC-61 relative to SCC-61 cells. The states of edges in the signaling subnetwork are also represented as binary variables (“1”: occurred or “0”: not occurred). The metabolic enzymes are similarly normalized to binary variables (“1”: activation or “0”: inactivation), which denote the corresponding metabolic reactions as blocked or unblocked. Our developed mathematical constraints were applied to describe: (1) the states of all the species (nodes) in this integral network; (2) the relationships between upstream and downstream proteins in the network topology; (3) the flux balance analysis (FBA) for ensuring the total amount of any compound being produced must be equal to the total amount being consumed at steady state; (4) the direction of net flux of each metabolic reaction is opposite to the change of Gibb’s free energy; and (5) the concentration of products in a single metabolic reaction is restricted by the state of the enzyme. The detailed information can be found in **Supplementary Material**. We named this mathematical framework as constraint-based systemic MIP for modeling redox-regulated networks (COSM^ro^).

**Figure 2:**
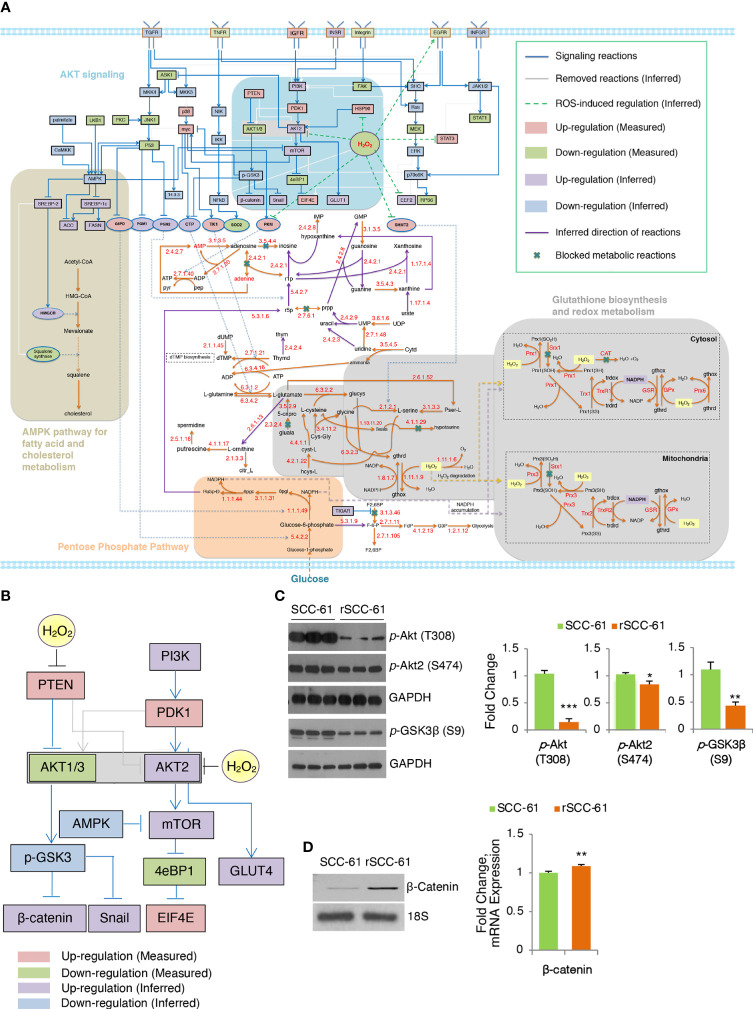
Inferred COSM^ro^ network differentiating radiation resistant rSCC-61 from radiation sensitive SCC-61 Cells. **(A)** rSCC-61 specific network inferred by COSM^ro^ model. Measured and inferred activity of signaling proteins and inferred direction of metabolic reactions is presented with four radiation resistant network signatures highlighted. **(B)** Akt subnetwork showing predicted upregulation of Akt2 and β-catenin and downregulated pGSK3β in radiation resistant rSCC-61 cells. **(C)** Western blot analysis of SCC-61 and rSCC-61 cell lysates with antibodies against pAkt (T308), pAkt2 (S474), pGSK3β (S9), and GAPDH as a loading control to validate network prediction. Quantification is shown on the right panels. **(D)** RT-PCR analysis showing increased expression of β-catenin in rSCC-61 compared to SCC-61 (left) and mRNA data extracted from the previously reported Illumina transcriptomic data (right). Primers are summarized in **Supplementary Table S4**. *p* values *0.05-0.01, **0.01-0.001, ***<0.001 (n = 3).

The COSM^ro^ formulation was solved with the MATLAB optimization toolbox Gurobi 9.0 (18) to guarantee minimal differences between measurements and predicted data and complexity of inferred network topology using the function in **Supplementary Material**, Formula 1. In the signaling subnetwork, the fitting precision of the optimized topology on 27 measured proteins is 96.3% (only the prediction for 14-3-3 protein was inconsistent). There were 16 signaling reactions (gray lines) removed from the generic network after optimization because the inferred states of these reactions indicated that they did not occur. In the metabolic subnetwork, 9 metabolic reactions are marked “×”, indicating there is decreased net flux passing through these biochemical reactions (**Figure 2A**). Significantly downregulated enzyme expression in the measured data leads to blockade of the reaction in the cell-specific pathways causing substrate accumulation and downregulation of all downstream metabolites (Constraint 10, **Supplementary Material**; **Figure S5D**). The direction of unblocked reversible reactions was inferred and marked with purple arrows. The predicting error of metabolic concentration of 38 differential metabolites was 0.4466.

The network analysis highlighted potentially interlinked pathway modules contributing to radiation resistance in HNSCC including (1) increased Akt2 and decreased Akt1/3 activities driving glucose uptake and activation of glycogen synthase kinase (GSK3) α/β signaling, (2) glycolytic flux diverted into PPP leading to an increase in precursors for nucleic acid biosynthesis and increased nicotinamide adenine dinucleotide phosphate (NADPH) production, (3) suppression of H_2_O_2_ in rSCC-61 through the enzymatic activities of cytosolic and mitochondrial peroxiredoxins, the NADPH-dependent thioredoxin/thioredoxin reductase (Trx/TrxR), and the glutathione reductase (GR) antioxidant systems, and (4) cholesterol metabolism (**Figure 2A**). These predicted outcomes from network analysis were further validated revealing the critical function of integrated redox signaling and metabolism in the response to ionizing radiation.

### Increased glucose uptake and GSK3 activities in rSCC-61 cells are determined by differential Redox regulation of Akt2 and Akt1/3

Redox activation of PI3K/Akt signaling through oxidative inactivation of its negative regulator PTEN is well established and it was included in COSM^ro^ network analysis (**Figure 2B**). Consistent with the increased PTEN activity in rSCC-61 resulting from lower ROS levels in this cell line, the overall phosphorylation of Akt isoforms was decreased in rSCC-61 (**Figure 2C** and **Figure S3B**). However, Akt2 phosphorylation was similar between SCC-61 and rSCC-61 cells. Given the increased H_2_O_2_ in SCC-61 (3) and the isoform-selective inhibition of Akt2 activity by H_2_O_2_ (19), the Akt2 activity was predicted to be upregulated in rSCC-61 (**Figure 2B**). Akt2 is a known regulator of glucose uptake by promoting glucose transporter (e.g., GLUT1) localization at the cell membrane (20). Our previously published data showed both increased membrane localization of GLUT1 and increased glucose uptake in rSCC-61 cells consistent with Akt2 activity predicted by the COSM^ro^ model (6). Since all Akt isoforms can phosphorylate GSK3β, the prediction was that overall GSK3β phosphorylation would be decreased in rSCC-61 driven primarily by the reduced activity of Akt1/3. Western blot analysis confirmed ~50% decreased GSK3β phosphorylation in rSCC-61 compared with SCC-61 (**Figure 2C**). GSK3α/β also mediates phosphorylation signaling regulating β-catenin stability and activation (21). COSM^ro^ predicted upregulation of β-catenin due to decreased GSK3β phosphorylation. Semi-quantitative PCR and mRNA data extracted from the previously reported Illumina transcriptomic data (4) show increased expression of β-catenin in rSCC-61 cells (**Figure 2D**).

### Increased NADPH Levels are driven by PPP and 1-C metabolism enzymes in rSCC-61 cells

**Figure 3:**
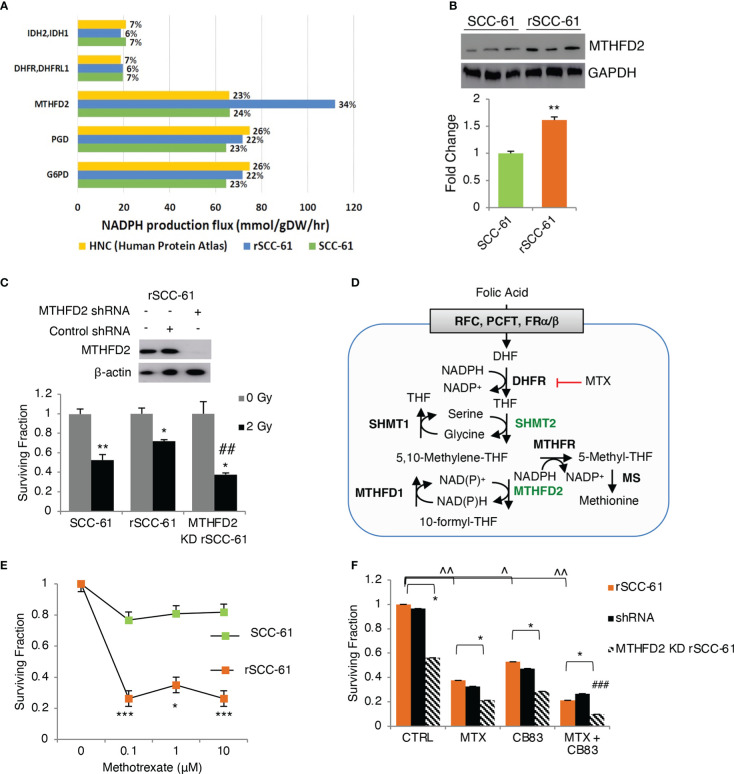
Flux balance analysis predicts MTHFD2 as key contributor to NADPH levels in radiation resistant cells. **(A)** Flux balance analysis of rSCC-61 and SCC-61 cells and HPV-negative HNSCC data extracted from the Human Protein Atlas identifies 7 genes: MTHFD2, PGD, G6PD, DHFR/DHFRL1 and IDH2/3 as contributing 84-90% of cellular NADPH production. **(B)** Western blot analysis confirms increased MTHFD2 expression in rSCC-61 cells compared to SCC-61 cells. **(C)** (upper) Western blot analysis of MTHFD2 in rSCC-61, rSCC-61 control shRNA and MTHFD2 shRNA lysates confirms selective knockdown (KD) of MTHFD2 in rSCC-61 cells; (lower) Clonogenic assay after 2 Gy irradiation of SCC-61, rSCC-61 and MTHFD2 KD rSCC-61 cells showing MTHFD2 depletion sensitizes radiation resistant cells to irradiation (*denotes statistical significance of radiation response relative to untreated cells for each group, and ^##^denotes statistical significance of radiation response in MTHFD2 KD rSCC-61 cells relative to rSCC-61 control shRNA; *p* values * 0.01-0.05 and ^##^ 0.01-0.001, n=3). **(D)** Schematic of 1-C metabolism contribution to NADPH. **(E)** Clonogenic assay showing treatment with 0 – 10 μM methotrexate (MTX) decreases rSCC-61 cell survival more significantly than SCC-61 cells. Statistical significance is relative to untreated control (*p* values * 0.01-0.05, *** <0.001, n=3). None of the SCC-61 treated conditions are significant compared to untreated control. **(F)** Clonogenic assay in rSCC-61, rSCC-61 control shRNA and MTHFD2 KD rSCC-61 demonstrating decreased cell survival by treatment with 0.1 μM MTX and 10 μM CB83 (G6PD inhibitor) and MTX and CB83 combined. MTHFD2 depletion decreased survival of rSCC-61 alone and in combination with MTX and/or CB83 inhibitors. ^###^ denotes the statistically significant decrease in cell survival (*p* 0.00063, n=3) with the knockdown of MTHFD2 and cotreatment with MTX and CB83 inhibitors. *p* values *,^ 0.01-0.05, **,^^ 0.01-0.001, ^###,^ ^^^<0.001 (n=3).

The COSM^ro^ prediction of increased PPP metabolic flux in rSCC-61 was consistent with our previously published energy metabolism studies showing decreased ATP synthesis and basal respiration in rSCC-61 (6). To build on these findings and quantify the relative contribution of PPP to NADPH reserve in SCC-61 and rSCC-61 cells, we generated genome-scale metabolic models predictive of flux contributions to NADPH production and consumption from the framework of the Human Metabolic Reaction (HMR) 2.0 model (22), as described in **Supplementary Material**. The SCC-61/rSCC-61 specific models were generated by estimating relative changes in metabolic gene transcriptomic data to populate upper flux constraints by the theoretical limit of Vmax values, as described in (5). The resulting FBA models containing 6,789 (SCC-61) and 6,596 (rSCC-61) reactions and compared to a catalog of 196 HPV-negative HNSCC samples (23) for the ability to maximize NAD(P)H production. From this analysis, we identified a subset of 7 genes shown in **Figure 3A** that are responsible for 84-90% of the cellular NAD(P)H production in the rSCC-61 and SCC-61 cells and thus will have the most significant impact on the suppression of ROS and response to radiation. Out of this, the PPP enzymes, glucose-6-phosphate dehydrogenase (G6PD) and 6-phosphogluconate dehydrogenase (PGD), collectively contributed 44% and 46% to the NADPH reserve in rSCC-61 and SCC-61 cells, respectively (**Figure 3A**).

#### 1-C metabolism and NAD(P)H reserve

The FBA also revealed the mitochondrial 1-C metabolism enzyme, MTHFD2, as a pronounced discriminator between cell lines in producing NAD(P)H with MTHFD2 contributing 34% and 24% in rSCC-61 and SCC-61, respectively (**Figure 3A**). The increased MTHFD2 protein in rSCC-61 was confirmed by Western blot analysis (**Figure 3B**). Clonogenic survival assays comparing the response to radiation in SCC-61, rSCC-61 and rSCC-61 with MTHFD2 knockdown further confirmed the function of this enzyme in the radiation resistance phenotype of rSCC-61 (**Figure 3C**). Together with our previously reported proteomics studies showing increased cytosolic MTHFD1 and mitochondrial SHMT2 in rSCC-61 by 6.3- and 4.0-fold, respectively, these studies emphasized the critical contribution of 1-C metabolism to the NAD(P)H reserve and response to radiation treatment (3, 24).

Methotrexate (MTX), an inhibitor of 1-C metabolism enzyme dihydrofolate reductase (DHFR/DHFRL1) is FDA-approved for the palliative care of HNSCC patients and often investigated in combination with other treatments to improve therapeutic efficacy (25). DHFR catalyzes the recycling of dihydrofolate byproduct of dTMP synthesis to THF in an NADPH-consuming reaction (**Figure 3D**) and its inhibition is expected to result in accumulation of cytosolic NADPH and depletion of the THF pool and the flux through mitochondrial SHMT2 and MTHFD2. This infers coordinated maintenance of cellular NAD(P)H between cytosolic and mitochondrial compartments, which could be targeted to maximize the build-up of cellular ROS. Thus, we investigated the clonogenic survival of SCC-61, rSCC-61, and MTHFD2 knockdown rSCC-61 cells treated with MTX and CB83, an irreversible inhibitor of the PPP rate-limiting enzyme G6PD (26). While each treatment condition decreased survival of rSCC-61 cells, the combined targeting of MTHFD2, DHFR and G6PD produced the most robust effect (<10% cell survival) (**Figures 3E, F**).

### Glutathione biosynthesis is optimized to increase H_2_S production in rSCC-61 cells

**Figure 4:**
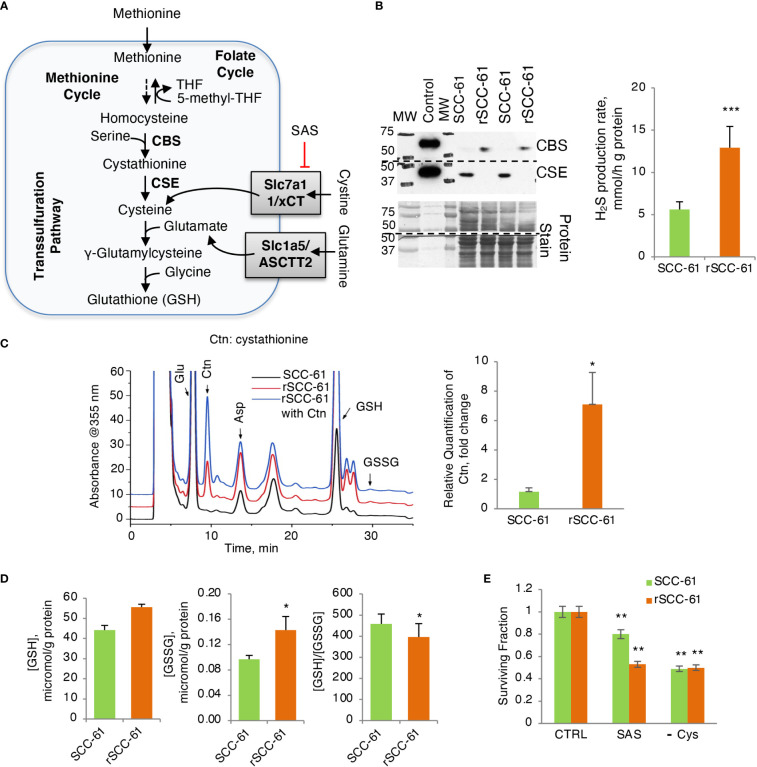
Increased H_2_S Production Rate in Radiation Resistant Cells. **(A)** Schematic of glutathione biosynthesis and cystine/glutamine transport in cells. **(B)** (Left) Western blot analysis of CSE and CBS enzymes showing reciprocal expression pattern in SCC-61 and rSCC-61 cells. Protein stain is shown as loading control and recombinant proteins were included as positive control. The blot was cut at 50 kDa and the upper part was used for quantification of CBS and the lower part for CSE. The MW denotes the position of proteins in the MW ladder copied to film by black marker. (Right) Measurement of H_2_S production rate showing increased capacity for H_2_S production in rSCC-61 cells (*p* value ***<0.001, n = 3). **(C)** HPLC analysis and mass spectrometry measurement of cystathionine accumulation in rSCC-61 compared to SCC-61 cells (*p* value *0.05-0.01, n = 3). **(D)** Analysis of GSH and GSSG showing increased GSH and GSSG but decreased GSH/GSSG in rSCC-61 compared to SCC-61 cells (*p* value *0.05-0.01, n = 3). **(E)** Decreased clonogenic survival in rSCC-61 cells treated with 0.5 mM of xCT inhibitor sulfasalazine (SAS) compared to SCC-61 cells. Depletion of cysteine in the media decreased survival of both cell lines by ~ 50%. Statistical significance is relative to control for each respective cell line (*p* value **0.01-0.001, n = 3).

The 1-C metabolism and PPP pathways contributing to the NADPH/NADP^+^ ratio are intrinsically connected to the levels and the balance of GSH and GSSG in cells (**Figure 4A**). GSH biosynthesis is rate limited by cysteine availability, which in turn is metabolically linked to 1-C metabolism and methionine cycle through homocysteine. NADPH is also a critical cofactor for the reduction of GSSG to GSH by Glutathione Reductase (GR) ultimately controlling the GSH/GSSG ratio in cells.

Western blot analysis of GSH biosynthesis enzymes identified cystathionine β-synthase (CBS) being expressed at higher level in rSCC-61 compared with SCC-61 cells and cystathionine γ-lyase (CSE) having the complementary profile with higher level in SCC-61 cells relative to rSCC-61 cells (**Figure 4B**), suggesting impaired H_2_S and/or GSH biosynthesis in both cell lines. Decreased CSE in rSCC-61 cells was correlated with cystathionine accumulation (**Figure 4C**), while the higher H_2_S production capacity in rSCC-61 cells was consistent with the CBS expression profile (**Figure 4B**). We also measured oxidized and reduced GSH to confirm earlier published results (3). Consistent with previous findings, higher levels of GSH and GSSG were found in rSCC-61 compared with SCC-61 cells (**Figure 4D**). To test whether the cystine uptake from the media could bypass a nonfunctional transsulfuration pathway in these cells, we determined cell survival after inhibiting the cystine-glutamate antiporter xCT with sulfasalazine (SAS) or alternatively growing cells in Cys depleted media. Inhibition of xCT as well as depletion of Cys in growth media led to a significant and comparable decrease in cell survival of both SCC-61 and rSCC-61 cells demonstrating the partial reliance of both SCC-61 and rSCC-61 cells survival on extracellular cystine pool (**Figure 4E**). Interestingly, rSCC-61 cells were more sensitive to SAS treatment compared with SCC-61 cells, while both cell lines were equally sensitive to Cys depletion.

### Fatty acids and cholesterol metabolism are independently regulated downstream of AMPK

**Figure 5:**
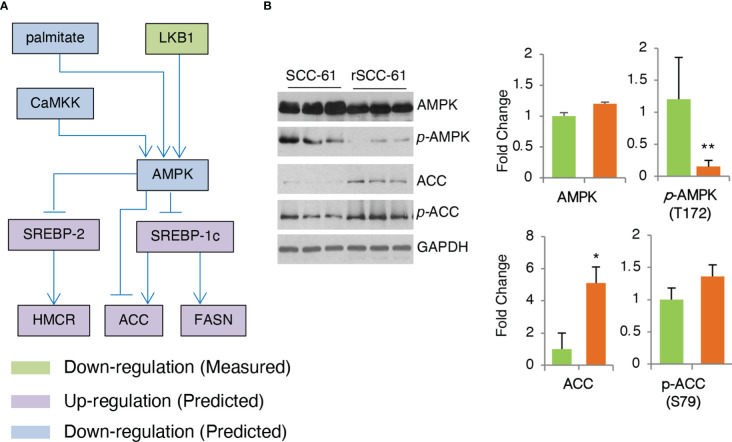
Validation of COSM^ro^ Subnetwork Connecting AMPK Activity to Fatty Acids and Cholesterol Metabolism. **(A)** Inferred network predicting down-regulated AMPK and upregulated ACC, FASN in rSCC-61 cells. **(B)** Western blot analysis of SCC-61 and rSCC-61 cell lysates for AMPK, pAMPK (T172), ACC and pACC (S79), and GAPDH used as a loading control. Quantification is shown on the right panels (*p* values *0.05-0.01, **0.01-0.001, n=3).

**Figure 6:**
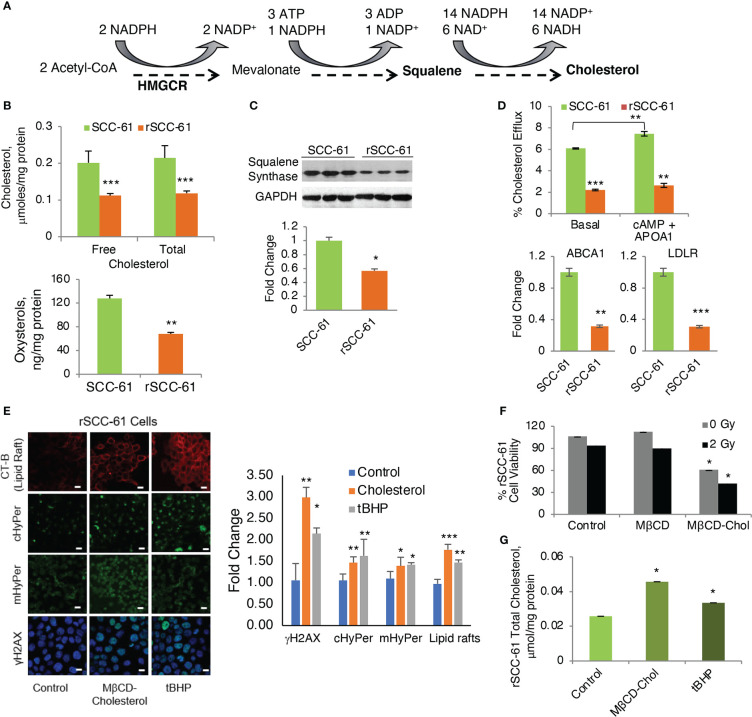
Regulation of Cholesterol and Its Impact on the Response to Radiation. **(A)** Abbreviated schematic of cholesterol biosynthesis showing the key NADPH consuming reactions. **(B)** Quantification of cholesterol and oxysterols by mass spectrometry showing significantly decreased cholesterol and oxysterols in rSCC-61 cells (*p* values **0.01-0.001, ***<0.001, n=3). **(C)** Western blot analysis of squalene synthase showing decreased expression in rSCC-61 compared to SCC-61 cells. Quantification is shown in the lower panel (*p* value *0.05-0.01). **(D)** Cholesterol efflux and uptake showing (upper) basal and maximal efflux in SCC-61 and rSCC-61 cells after treatment with cAMP and human APOA1, and (lower) Relative quantification of ABCA1 responsible for the efflux of free cholesterol and LDLR involved in the uptake of cholesterol esters in SCC-61 and rSCC-61 cells show both their gene transcript levels to be lower in rSCC-61 cells (*p* values **0.01-0.001, ***<0.001, n=3). **(E)** (left) Fluorescence imaging of lipid rafts with cholera toxin-beta (CT-B), cytosolic and mitochondrial H_2_O_2_ production with HyPer and DNA damage with γH2AX in rSCC-61 cells after treatment with 1 mM MβCD-cholesterol complex and 1 mM tBuOOH showing increased lipid rafts, H_2_O_2_, and nuclear γH2AX foci formation (scale bar: 100 μm). Quantification is shown on the right panel (*p* values *0.05-0.01, **0.01-0.001, ***<0.001, n=3). **(F)** MTT assay of cell viability of rSCC-61 cells at 48h after treatment 1 mM MβCD-cholesterol complex and 2 Gy irradiation demonstrating increased sensitivity to radiation (*p* values *0.05-0.01, n=3). **(G)** Increased total cholesterol in rSCC-61 cells treated with 1 mM MβCD-cholesterol complex or 1 mM tBuOOH (*p* value *0.05-0.01, n=3).

AMPK is a critical enzyme in the cellular response to metabolic stress regulating lipid, cholesterol and glucose metabolism (27). COSM^ro^ model predicted AMPK downregulation in the rSCC-61 cells (**Figure 5A**). This was confirmed by Western blot analysis showing decreased phosphorylation of AMPK in rSCC-61 cells resulting in upregulation of critical enzymes involved in lipogenesis including acetyl-CoA carboxylase (ACC) (**Figure 5B**) and fatty acid synthase (FASN) (6). AMPK-deficient cells would be expected to exhibit enhanced rates of lipid and cholesterol synthesis through mechanisms involving ACC and 3-hydroxy-3-methylglutaryl-CoA reductase (HMGCR) (28), the expression of which is controlled by sterol regulatory element-binding protein SREBP1c and SREBP2, respectively (**Figure 6A**). We previously reported increased FASN and utilization of endogenous fatty acid β-oxidation for energy production in rSCC-61 cells (6) consistent with the COSM^ro^ predictions.

Contrary to the expectation based on AMPK activity, COSM^ro^ analysis predicted decreased cholesterol in rSCC-61 (**Figure 2A**). Indeed, when we measured cholesterol and oxysterol derivatives with mass spectrometry, we found reduced levels of these species in rSCC-61 (**Figure 6B**) consistent with the model predictions, which we rationalized was driven by the ~15-fold higher squalene measured in rSCC-61 (**Supplementary Dataset S1**). Interestingly, however, Western blot analysis revealed lower squalene synthase in rSCC-61 cells (**Figure 6C**) suggesting either posttranslational impairment of squalene synthase activity in SCC-61 or differential cholesterol uptake and efflux in the two cell lines.

As the COSM^ro^ model did not account for cholesterol transport across cell membranes, we investigated experimentally the potential contribution of cholesterol uptake and efflux to the decreased cholesterol in rSCC-61 cells. A higher rate of cholesterol efflux and/or lower cholesterol uptake in rSCC-61 could also explain the decreased intracellular cholesterol in this cell line. The ATP binding cassette subfamily A member 1 (ABCA1) is a major regulator of cellular cholesterol efflux, while the low-density lipoprotein receptor (LDLR) controls cholesterol uptake. Gene expression data showed a decreased level of both ABCA1 and LDLR proteins in rSCC-61 cells (**Figure 6D**, lower panel). Consistent with this analysis, we found lower basal cholesterol efflux in rSCC-61 cells relative to SCC-61 cells and slight increases in ABCA1-mediated cholesterol efflux for both cell lines relative to basal efflux though this was statistically significant only for SCC-61 cells (**Figure 6D**, upper panel). Altogether, these data show potentially slower kinetics of cholesterol uptake and efflux in rSCC-61 cells, they do not conclusively support cholesterol trafficking as the cause for decreased cholesterol levels in rSCC-61 cells. In addition to differences in LDLR, the mRNA expression data showed downregulation of other SREBP2 target genes in rSCC-61 such as HMGCR (**Figure S6A**), while COSM^ro^ model predicted upregulation of the HMGCR activity. Decreased expression of HMGCR is consistent with squalene synthase data and is expected as both genes are regulated by SREBP2. Thus, despite the predicted activation of SREBP2 by AMPK, SREBP2 activity in rSCC-61 is decreased. This could be due to impaired SREBP2 translocation to the nucleus in rSCC-61 cells a hypothesis supported by the cumulative action of insulin induced genes (INSIG1/2) and 24-OH oxysterol, which are both significantly higher in rSCC-61 (**Figure S6B**). Alternatively, SREBP2 escort to the Golgi by SCAP may be impaired, resulting in reduced processing of SREBP2 in the Golgi. This remains to be explored in future studies.

### The interdependence of NADPH and cholesterol metabolism is mediated by steroidogenesis

**Figure 7:**
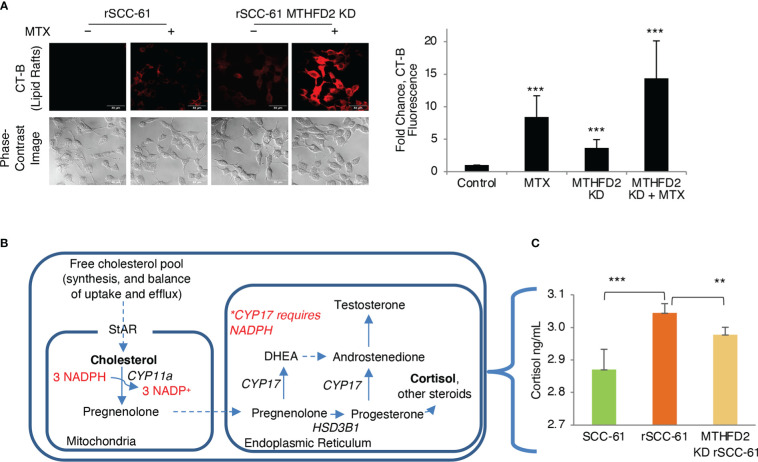
Regulation of lipid rafts and steroidogenesis by MTHFD2. **(A)** Knockdown of MTHFD2 in rSCC-61 cells increases lipid rafts and the effects are further enhanced by inhibition of dihydrofolate reductase (DHFR) by methotrexate (MTX) (scale bar 50 μm). Quantification is shown on the right panel (*p* values ***<0.001, n=3). **(B)** Abbreviated schematic of steroidogenesis highlighting the requirement for NADPH in the mitochondria to support CYP11a activity. **(C)** Measurement of cortisol in cell extracts showing statistically significant higher cortisol in rSCC-61 relative to SCC-61 cells, and attenuation of its levels in MTHFD2 KD rSCC-61 cells. (*p* values **0.01-0.001, ***<0.001, n=3).

We have reported the interdependence of H_2_O_2_ and lipid rafts in SCC-61 cells in previous studies (3). Here, we performed complementary treatments by increasing intracellular ROS and cholesterol in rSCC-61 cells both of which promoted lipid raft formation. We found increased cholesterol and lipid rafts led to more H_2_O_2_ in the cytosol and mitochondria resulting in increased DNA double strand breaks as indicated by imaging with γH2AX (**Figure 6E**). This change in redox state mediated by lipid raft formation led to the sensitization of rSCC-61 to 2 Gy ionizing radiation (**Figure 6F**). We also found increased lipid rafts in rSCC-61 cells treated with *tert*-butyl hydroperoxide (tBHP), associated with higher intracellular cholesterol (**Figure 6G**). Higher intracellular ROS would be expected to deplete NADPH, and since cholesterol biosynthesis depends on the NADPH availability, these findings seemed counter-intuitive. Thus, we investigated the effects of MTHFD2 depletion on the lipid rafts content, and found a significant accumulation of lipid rafts which was further enhanced by treatment with MTX (**Figure 7A**).

To resolve this conundrum, we focused on the potential impact of MTHFD2 on steroidogenesis given the mitochondrial localization of both NADPH-producing MTHFD2 and NADPH-consuming CYP11a, the enzyme catalyzing the first step in cortisol and other steroids biosynthesis from cholesterol (**Figure 7B**). MTHFD2 knockdown in rSCC-61 was expected to result in inhibition of steroidogenesis and accumulation of cholesterol. Indeed, when cortisol was measured in SCC-61, rSCC-61 and MTHFD2 knockdown rSCC-61 cells, the rSCC-61 cells had higher cortisol compared with SCC-61 cells and this was decreased by knockdown of MTHFD2 (**Figure 7C**).

## Discussion

We present here a constraint-based systemic modeling of integrated redox signaling and metabolic networks (COSM^ro^) using multi-omics and complementary data (**Figure 1**). We combined enriched signaling and metabolic pathways to obtain a generic pathway map and then applied a MIP approach for joint optimization of signaling and metabolism to infer signaling and metabolic networks that define the rSCC-61 radiation resistance phenotype. The MIP approach generated 412 constraints (115 quadratic constraints and 297 linear constrains) and 502 variables in the optimization process of the pathways map resulting in removal of 15 signaling reactions from the generic network due to inconsistency in the states of connected proteins and of 9 metabolic reactions predicted as being “blocked”. The optimized network topology in **Figure 2A** fits the measured data very well. Compared to other systems modeling approaches, our method, which is based on constraints, can simplify the optimization process and quickly search an optimal solution in an allowable subspace. Given the details of the system analyzed here – large scale of generic pathways, a single measured data point – ratio of rSCC-61 to SCC-61 – an ODE-based method could not have been suitable for analysis (9).

Integrated COSM^ro^ analysis discovered critical functions of redox-regulated signaling and metabolic pathways defining the rSCC-61 phenotype. We validated the predicted differential regulation of Akt isoforms and its downstream targets using Western blot and semi-quantitative PCR (**Figure 2B**). COSM^ro^ predicted increased Akt2 activity in rSCC-61 cells due to more reducing environment in this cell line. This prediction is consistent with increased glucose uptake in rSCC-61 reported in an earlier publication (6). On the other hand, Akt1/3 phosphorylation was decreased in rSCC-61 cells resulting in decreased phosphorylation of downstream target GSK3β and upregulation of downstream β-catenin (**Figure 2C**). GSK3β is a critical regulator of innate inflammatory processes and radiation-induced apoptosis. Thus, attenuation of GSK3β in rSCC-61 cells suppresses the expression of NF-κB mediated pro-inflammatory genes, which were shown to protect against radiation-induced cell death (29).

COSM^ro^ analysis also considered the contribution of KEAP1/Nrf2 antioxidant system with special emphasis on evaluating the NADPH as electron donor for suppression of oxidation. NADPH provides the reducing equivalents necessary for the regeneration of GSH by GR and activity of NADPH-dependent TRX/TR systems and supports the activity of peroxiredoxins and glutathione peroxidase. The COSM^ro^ model included targets of KEAP1/Nrf2 signaling such as xCT, GCLC, GR, GPX, TXN, TXNR, SRX, G6PD, PGD and others. To better understand and quantify NADPH-producing pathways, we performed complementary FBA to identify the key metabolic enzymes contributing to the NADPH reserve in SCC-61 and rSCC-61 cells. The analysis revealed the 1-C metabolism mitochondrial enzyme, MTHFD2, as a key upregulated NADPH-producing enzyme in rSCC-61 compared to SCC-61 cells, which was then further investigated *in vitro* and *in vivo* (24). To further delineate the role of MTHFD2 in rSCC-61 cells, we knocked out MTHFD2 in these cells, which resulted in a 40% reduction in cell survival after irradiation (**Figure 3C**). MTHFD2 knockdown in combination with targeted inhibition of DHFR and G6PD significantly decreased clonogenic survival of rSCC-61 to < 10% compared to SCC-61 cells (**Figure 3F**). Altogether, these data demonstrate compensatory mitochondrial 1-C metabolism and cytosolic PPP to maintain cellular NADPH. As evident by singular inhibition of PPP or 1-C enzymes, uncoupling of metabolic coordination resulted in lesser inhibitory effects on cell survival and proliferation in rSCC-61 cells.

As NADPH is intrinsically connected to the levels of reduced GSH in cells through the activity of GR, we sought to determine if the GSH/GSSG ratio shifted as well towards a more reduced state in rSCC-61. Interestingly, quantitative analysis showed increased synthesis of GSH with higher levels of both GSH and GSSG in rSCC-61, but only slight increase in the GSH/GSSG ratio (**Figure 4D**). This was surprising as both cell lines were deficient in one of the key GSH biosynthesis enzymes: CBS in SCC-61 and CSE in rSCC-61 (**Figure 4B**). Potential reasoning for the data lies in earlier studies in colon cancer showing differential effects of CBS and CSE (30). Lower CBS activity was linked to reduced glycolytic functions and ATP production as well as increased ROS levels compared to no change in these parameters after silencing CSE. Our results are consistent with these data. SCC-61 cells are characterized by lower CBS, decreased glycolysis and increased ROS. Other studies suggest H_2_S may contribute to tumor growth through activation of PI3K/Akt pathway, inhibition of phosphatases or by regulating expression of cell cycle genes (31, 32). Increased H_2_S production and higher Akt phosphorylation in rSCC-61 are consistent with these findings. We further identified a dependence of both SCC-61 and rSCC-61 cells on cystine import to support glutathione biosynthesis as CBS and CSE enzymes seem to be utilized primarily for production of H_2_S (**Figure 4B, E**).

Our published energy metabolism studies showed increased funneling of glycolytic intermediates into the PPP in rSCC-61 cells resulting in decreased mitochondrial oxidative phosphorylation activity and reduced mitochondrial ROS (6). COSM^ro^ predicted downregulation of AMPK, a key sensor of cellular energy, resulting in increased SREBP activity, and upregulation of fatty acid and cholesterol biosynthesis in rSCC-61 cells (**Figure 5**). Indeed, AMPK downregulation was consistent with significantly upregulated FASN in rSCC-61 cells. However, LC-MS/MS analysis found decreased cholesterol and oxysterols but increased squalene in rSCC-61 cells contradictory to the established paradigm in the literature (**Figure 6B**, **Supplementary Dataset S1**). While squalene is a known antioxidant and could be upregulated to provide defense against oxidative stress, its association with radiation resistance has not been explored. Decreased cholesterol could be due to downregulation of cholesterol biosynthesis enzymes downstream of squalene (controlled by SREBP2) or by their posttranslational regulation. Indeed, expression of key SREBP2 downstream genes (e.g., LDLR, HMGCR) is lower while expression of SREBP2 inhibitory genes (e.g., INSIG1, AMFR) is higher in rSCC-61 compared to SCC-61 cells (**Figure S6B**) bringing support to regulation at the level of gene expression. Cholesterol uptake and efflux data ruled out the deficiency in cholesterol transport across the cell membrane (**Figure 6D**). The matched cell model system of acquired resistance to radiation employed here ultimately revealed non-adrenal steroidogenesis and redox metabolism, particularly the enzymes controlling mitochondrial NADPH, as potential targets for cancer treatment (**Figure 7**). Previous cancer research on steroidogenesis has focused primarily on classical steroidogenic tissues (e.g., ovarian and testicular cancer) and a limited number of other tissues such as brain, gut, heart, prostate and skin where steroidogenesis was shown to occur and impact both local and systemic immunity. This is the first report showing active steroidogenesis in HNSCC and its dependence on mitochondrial 1-C metabolism enzyme MTHFD2.

In summary, the work presented here describes a new mathematical approach to account for redox effects in signaling and metabolism, COSM^ro^, which when combined with complementary flux balance analysis identified several key, but yet unexplored metabolic mechanisms of resistance to radiation treatment, which include increased capacity for H_2_S production and increased steroidogenesis in radiation resistant cells. These model-based predictions need to be validated further using human HNSCC specimens and/or mouse xenograft models.

## Materials and methods

### Generation and primary characterizations of SCC-61/rSCC-61 matched model system of radiation resistance in HNSCC

We have reported the establishment of the radiation resistant rSCC-61 cell line in a previous publication (3). Briefly, the radiation sensitive SCC-61 cells were irradiated using a 2 Gy radiation dose. After the radiation treatment the cells were cultured, split, allowed to achieve 60% confluence and then exposed to another cycle of 2 Gy radiation. This process was repeated for cumulative total of 16 Gy. The resulting cell population was plated at low density on soft agar. rSCC-61 was picked as a single colony, expanded in culture and profiled using genomics (4), proteomics methods (3), and complementary methods (6).

### Quantitative redox proteomics

SCC-61 cells and rSCC-61 cells were cultured in DMEM/F12 media containing the light and heavy isotopes of Lys8 and Arg10, respectively, and supplemented with 10% dialyzed FBS and 200 mg/L proline to prevent the conversion of isotope-coded arginine to proline in cells (33). The cells were then lysed in 0.5 mL with modified RIPA buffer (50 mM Tris-HCl, pH 7.4; 1% NP40; 15 mM NaCl; 1 mM EDTA; 1 mM NaF; Roche protease and phosphatase inhibitor tablets) supplemented with 1 mM 1,3-cyclopentanedione (BP1; biotin-tagged selective protein sulfenylation reagent; Xoder Technologies) and 200 U/mL catalase. Protein concentration was determined using BCA assay (Thermo Scientific). Samples were normalized, combined 1:1, precipitated by cold acetone and pellets were resuspended in minimum volume (approx. 100 μL) of 1% SDS in PBS. Samples were diluted 10-fold prior to enrichment with 50 mM ammonium bicarbonate. Approximately 250 μL of suspended streptavidin agarose beads (Cat. # 20361, Thermo/Invitrogen) were used for each 1 mg of lysate. The beads were equilibrated 3 times with 4 bed volumes of 0.1% SDS in PBS for 10 min each. Biotinylated lysates were added to the beads and incubated on rotator (end-over-end) overnight at 4 °C. The non-specifically bound proteins were removed by washing the beads sequentially with 2 M urea, 1 M NaCl, 0.1% SDS, 10 mM DTT, and 50 mM ammonium bicarbonate. The proteins on beads were digested overnight at 37°C on a shaker with trypsin using a 1:50 enzyme-to-substrate ratio. The resulting peptides were acidified with 1% formic acid, centrifuged at 2,000 g for 5 min, and desalted using a tC18 SepPak column (Cat #WAT036820) following the manufacturer’s protocol. The dried samples were used in HPLC fraction immediately or stored in -20°C.

#### HPLC fractionation

Basic high pH reversed-phase chromatography was conducted on a Waters 2695 instrument using a Xbridge C18, 3.5 µm, 4.6 x 150 mm chromatography column. Desalted peptides were reconstituted in 20 mM ammonium formate, pH 10, injected and separated using a flow rate of 0.5 mL/min and the following gradient: Solvent A (2% acetonitrile (ACN), 5 mM ammonium formate, pH 10), Solvent B (90% acetonitrile, 5 mM ammonium formate, pH 10); 0% B for 2 min; 0-10% B in 5 min; 10- 27% B in 34 min; 27- 31% B in 4 min; 31- 39% B in 4 min; 39-60% in 7 min; 60% B for 8 min; 60-100% B in 2 min; 100% B for 4 min; 100-0% B in 1 min; 0% B for 1 min]. Eluted peptides fractions (1 mL/tube) were collected using Water Fraction Collector III. The 36 eluted fractions were combined from the beginning, middle, and end of the run to generate 12 fractions (1,13,25; 2,14,26; etc) and then dried using a SpeedVac Savant™ SPD1010 (Thermo).

#### NanoLC MS/MS and database searching

Dried peptide extracts were dissolved in 0.1% formic acid, 5% ACN, and separated by nanoLC (Dionex Ultimate 3000) equipped with a Nano Trap Column, Acclaim PepMap100 (C18, 5 µm, 100Å, 100 µm i.d. x 2 cm nanoViper) and an Acclaim PepMap RSLC nanocolumn (C18, 2 µm, 100 Å, 75 µm i.d. x 15 cm, nanoViper) (Thermo Scientific). The samples (10 µL) were injected and separated on the nanocolumn at a flow rate of 300 nL/min using the following gradient: Solvent A: 95% water, 5% ACN, 0.1% formic acid; Solvent B: 20% water, 80% ACN, 0.1% formic acid); 2–10 min: 2–7% B; 10– 130 min: 7–50% B; 130–132 min: 50–85% B; 132–147 min: 85% B; 147-150 min: 85-2% B; 150–160 min, 2% B. LTQ Orbitrap Velos Pro (Thermo Scientific) was used for MS/MS analysis. The sample was ionized in the nanospray source equipped with stainless steel emitter (Thermo Scientific). The spray voltage was set to 1.5 kV and the temperature of the heated capillary was set to 200°C. Full scan MS survey spectra in profile mode were acquired in the Orbitrap. In positive ion mode by alternating full-scan MS 10 most intense peaks in the ion trap with dynamic exclusion enabled. These most intense peptide ions were fragmented by collision-induced dissociation (CID) using a normalized collision energy of 35.0. The resolution was 60,000. The lock mass (371.101240) option was enabled for survey scans to improve mass accuracy. Data were acquired using the XCalibur v.2.1 software. Protein identification and quantitation was performed using Proteome Discoverer v 1.4 and human protein database downloaded in FASTA format from UniProt (http://www.uniprot.org/). Data files (.raw format) were searched against the downloaded database using the following parameters: Enzyme: trypsin; Max. Missed Cleavage Sites: 2; Search Mode: MS/MS ion search with decoy database search included; Precursor Mass Tolerance: 10 ppm, Fragment Mass Tolerance: 0.6 Da; Target False Discovery Rate (FDR): 0.01.

### Metabolomics studies

Cells (10^8^/sample) were first extracted with 500 mL of mixture solvent (methanol:chloroform:water 2.5:1:1), followed by addition of 500 mL of methanol. The supernatant was transferred to gas chromatography (GC) sampling vials and vacuum dried at room temperature (RT). The residue was then derivatized using a two-step procedure. First, 80 μL methoxyamine (15 mg/mL in pyridine) was added to each vial and incubated for 90 min at 30°C. This was followed by addition of 80 μL BSTFA (N,O-bis(trimethylsilyl)trifluoroacetamide) (1% TMCS, trimethylchlorosilane) to initiate the derivatization reaction. The samples were heated for 60 min at 70°C and 1-μL aliquot of the derivatized solution was injected in splitless mode into an Agilent 7890N gas chromatograph coupled with a Pegasus HT time-of-flight mass spectrometer (TOF MS; Leco Corporation, St Joseph, USA). Separation was achieved on a Rxi-5MS capillary column (30 m × 250 µm I.D., 0.25-µm film thickness, Restek), with helium as carrier gas at a constant flow rate of 1.0 mL/min. The temperature of injection, transfer interface, and ion source were set to 260°C, 260°C, and 210°C, respectively. The GC temperature programming was set to 80°C for 2 min, followed by 10°C/min oven temperature ramps to 140°C, 4°C/min to 210°C, 10°C/min to 240°C, and 25°C/min to 290°C, and a final 4.5 min maintenance at 290°C. Electron impact ionization (70 eV) at full scan mode (m/z 40-600) was used, with an acquisition rate of 20 spectra/second in the TOF MS setting. The acquired MS files from GC/TOF MS analysis were analyzed by ChromaTOF software (v4.22, Leco Co., CA, USA). Statistic component analysis provided the aligned data sheet containing sample information, unique mass, and the area of unique mass of each sample. Internal standards and any known artificial peaks, such as peaks caused by noise, column bleed and BSTFA derivatization procedure, were removed from the data set. The resulting data was normalized to sum of all peaks prior to statistical analysis (and multiplied by 10,000). The normalized data was mean centered and unit variance scaled during chemometric data analysis in the SIMCA-p 12.0 Software package (Umetrics, Umeå, Sweden). Compound identification was performed by comparing the mass fragments with NIST 11 Standard mass spectral databases in NIST MS search 2.0 (NIST, Gaithersburg, MD) software with a similarity of more than 70%.

### General method for western blot analysis

Cells were lysed with the modified RIPA buffer as described above. The lysates were incubated on ice for 1 h followed by centrifugation at 13,000 x g for 10 min. The lysates were then normalized for their protein concentration across different treatment conditions and subjected to SDS-PAGE. The separated proteins were then transferred to a nitrocellulose membrane (0.45 µm, BioRad) and probed for the indicated proteins after overnight incubation with the corresponding primary antibodies diluted in 5% BSA in Tris Buffered Saline (TBS)-Tween20 buffer followed by incubation with respective HRP-conjugated secondary antibodies. The western blots were developed using Western Lightning Plus-ECL reagents followed by exposure to autoradiography film (Blue Ultra Autorad Film from GeneMate). The protein ratio in rSCC-61 vs. SCC-61 was quantified using ImageJ.

#### CBS and CSE western blot analysis

100 µg of total protein was loaded per each sample lane of 1 mm 10% gel and 5 ng of human cystathionine beta-synthase (CBS) and 30 ng of human cystathionase (CSE) were loaded as standards. Electrophoresis was run at 150V. Proteins were transferred to PVDF membrane during 90 min procedure at 25 V using Invitrogen XCell II Blot Module. After transfer the membrane was incubated in milk tris-buffered saline (TBS-T (tween-20) 40 min at RT to block unspecific binding and cut at the level of 50 kD. Upper part of the membrane was treated with anti-CBS, and lower part with anti-CSE antibodies overnight at 4°C. Both antibodies were chicken IgY (Aves Labs, Inc). Then membranes were washed out of primary antibodies and incubated with secondary antibodies (Horseradish peroxidase linked goat anti-chicken, Aves Labs Inc) diluted 1:250,000 for 1 h at RT. Then secondary antibodies were washed out, membrane was treated with Clarity Western ECL Substrate (Bio Rad) and exposed to film. After film development the membranes were stained with Ponceau S as recommended by Invitrogen for equal loading and transfer control.

### Quantitative analysis of intracellular GSH and GSSG

To measure the concentration of intracellular GSH and GSSG, 2 – 4 x 10^6^ cells were grown in a 60 mm culture dish. Cells were washed 3X with ice-cold PBS, then 0.5 mL of PBS was added to the culture plates on ice. Cells were detached by scraping and collected in a 1.5 mL centrifuge tube. An aliquot of the cell suspension in PBS was mixed with an equal volume of metaphosphoric acid solution (16.8 mg/ml HPO_3_, 2 mg/ml EDTA and 9 mg/ml NaCl) and vortexed gently. The precipitated proteins were separated by centrifugation at 13,000 x g for 10 min at 4°C. The supernatant was transferred into a 1.5 mL centrifuge tube, iodoacetic acid was added at a final concentration of 7 mM to alkylate free thiols, the pH was adjusted to 7 - 8 with saturated K_2_CO_3_ and the reaction was allowed to proceed for 1 h in the dark at RT. Finally, an equal volume of 2,4-dinitrofluorobenzene solution (1.5% v/v in absolute ethanol) was added to the mixture, then vortexed, and the reaction was allowed to proceed for at least 4 h in the dark at RT. The N-dinitrophenyl derivatives of GSH and GSSG were separated by HPLC on a Waters μBondapak NH2 column (300 mm x 3.9 mm, 10 μm) at a flow rate of 1 mL/min. The mobile phase consisted of solvent A (4:1 methanol/water mixture) and solvent B, which were prepared by mixing 154 g of ammonium acetate in 122 mL of water and 378 mL of glacial acetic acid and adding 500 mL of the resulting solution to 1,000 mL of solvent A. The following elution conditions were used: from 0 – 10 min isocratic 25% solvent B; from 10 – 30 min linear gradient 25 – 100% solvent B; from 30 – 34 min 100% solvent B; from 34 – 36 min 100 – 25% solvent B; and finally, 36 – 45 min 25% solvent B. Prior to injection, the column was equilibrated with 25% solution B for 10 min. Elution of metabolites was monitored by their absorbance at 355 nm. Under these conditions, GSH and GSSG exhibit retention times of 26 and 29 min, respectively. The concentration of individual thiols is determined by comparing the integrated peak areas with independently generated calibration curves for each compound. The concentration of each metabolite was normalized to the protein concentration measured by the Protein Assay Dye Reagent (Bio-Rad) in the corresponding sample. The detection limit of this method is ~ 20 pmol.

### Measurement of H_2_S producing capacity

SCC-61 and rSCC-61 cells were grown in 100 mm Petri dishes until ~80% confluence. Then the medium was aspirated, cells were washed two times with cold PBS and then trypsinized or scraped from the surface with small amount of PBS. Cell suspension was placed into preweighed sample tubes and centrifuged for 5 min at 2000 x g to sediment cells. The supernatant was aspirated and tubes were weighed again. Then 100 mM HEPES buffers, pH 7.4 was added to the tubes in a volume equal to 9 volumes of cell pellet assuming the cell density equal to 1 g/mL. Tubes were vortexed and freeze/thawed 3 times to lyse the cells. Protein levels in lysate were measured using Protein Assay Dye Reagent (Bio-Rad). To measure the rate of H_2_S production capacity, 400 μL of cell lysate was mixed with L-cysteine (Sigma) (20 mM final concentration) and D,L-homocysteine (Sigma) (40 mM final concentration) in a total volume of 500 μL and incubated under anaerobic conditions for 20 min at 37°C. Accumulation of H_2_S was measured using GC equipped with sulfur chemiluminescence detector (SCD 355) (Agilent) as described (34). Cell lysates alone as well as mixture of cysteine and homocysteine in buffer were used as control samples. H_2_S levels obtained in control samples were subtracted from H_2_S levels obtained in experimental samples. The rate of H_2_S production was normalized to the amount of protein in lysate.

### Clonogenic assays

rSCC-61 and SCC-61 cells were grown to 60% confluence. Cells were then trypsinized and resuspended in complete medium (DMEM/F12 + 10% FBS and 0.5% PenStrep (Gibco)), and plated (300 cells/well) into six well culture dishes. Following overnight incubation at 37°C, each 6-well plate was treated with either (i) 1 μM sulfasalazine (SAS) in media, (ii) media without cysteine, or (iii) 0 – 10 μM methotrexate and allowed to incubate further for 12 days for the colony formation. For other clonogenic assays, first the rSCC-61 cells were grown to 60% confluence followed by transfection with MTHFD2 shRNA (50 nM) or control shRNA (50 nM) (Santa Cruz Biotechnology) using Lipofectamine 2000 (ThermoFisher Scientific) according to manufacturer’s protocol. After 48 h, transfected cells were trypsinized, resuspended in complete medium (DMEM/F12 + 10% FBS and 0.5% PenStrep), and selected using puromycin. After selection, the cells were plated (300 cells/well) into six well culture dishes and incubated overnight. Cells were then subjected to (i) 2 Gy irradiation, (ii) 10 μM methotrexate, (iii) 1.7 μM CB83, or (iv) 0.1 μM methotrexate combined with 1.7 μM CB83 and incubated for an additional 12 days for the colony formation. Once formed, the colonies were fixed in methanol and acetic acid (7:1) solution and stained with 0.5% crystal violet (Fisher Scientific). The colonies containing more than 50 cells were counted. The surviving fraction of the treated cells was normalized using the plating efficiencies of their corresponding untreated control. The data were fitted to the multi-target and linear quadratic formulae, where survival (S) was related to dose (D) by the expression $S=1-{\left(1-\;e^{-\frac{D}{D0}}\right)}^{N\;}$ and *S*=*e*−^*αD*− *βD*2^ using SigmaPlot v.12 software (35).

### MTT cell viability assay

To assess the effects of lipid raft activation on response to radiation in rSCC-61 cells, the cells were trypsinized, resuspended in complete DMEM/F12 medium and seeded in 24-well plates at a density of 5 x 10^4^ cells/mL. After overnight incubation at 37°C, cells were treated with 1 mM MβCD and 1 mM MβCD-Cholesterol complex for 24 h followed by 2 Gy radiation for 48 h. After incubation, 0.125 mL MTT (5 mg/mL in PBS) was added to each well and plates were further incubated for 4 h at 37°C. At the end of incubation, 0.5 mL solubilization solution was added to each well and the absorbance was recorded at 570 nm.

### Cholesterol and oxysterols analysis

Cells were grown to ~70% confluence in complete DMEM/F12 media. Cholesterol and oxysterols were extracted from 1 x 10^6^ million SCC-61 or rSCC-61 cells using the procedures described next. For free cholesterol analysis, each cell pellet was extracted with 1 mL of ice-cold methanol to which 20 μL of oxysterol internal standard mixture (d_7_-24-OH-cholesterol, d_3_-25-OH-cholesterol, d_7_-7-ketocholesterol and d_5_-27-OH-cholesterol each at 1 ng/μL) and 30 μL of [^13^C_2_]-cholesterol (1 μg/μL) was added and then mixed by vortexing. Extracts were incubated on ice for 1 h before being centrifuged at 18,000 x g. The supernatant was removed and then stored at -20°C. Free cholesterol was measured in a 1:10 dilution of the extract in methanol. For total cholesterol analysis, total cholesterol was measured after saponification of 100 μL of the initial cold methanol extraction. The 100 μL subjected to saponification was first evaporated under nitrogen until dryness and reconstituted in 1 mL of ethanol to which 100 μL of 50% KOH was added. Each sample was then capped, flushed with argon, and incubated at 60°C for 1 hour. After incubation, 1 mL of water and 3 mL of hexane were added and the samples were mixed by vortexing. These samples were then centrifuged for 5 min at 3000 x g to separate the layers. The hexane layers were then removed and evaporated to dryness under nitrogen before being reconstituted in 1 mL of methanol. For oxysterol analysis, samples were evaporated under nitrogen and re-dissolved in methanol followed by vortexing and addition of 100 μL of aqueous 50% KOH (w/w). Samples were vortexed and incubated at RT for 2 h with vortexing every 30 min. Next, a 3:1 addition of toluene and distilled H_2_O were added to the sample followed by vortexing and 4000 rpm centrifugation for 5 min. The upper phase was evaporated under nitrogen and re-dissolved in toluene and vortexed thoroughly. Isolute 100 mg silica SPE-cartridges (Isolute, Cat #460-0010-A) were preconditioned with hexane and samples were loaded onto column in toluene. Columns were washed with 1 mL hexane, 8 mL hexane:isopropanol (99.5:0.5) and then the oxysterol fraction was eluted with 5 mL hexane:isopropanol (70:30). The analysis was performed on a Shimadzu UHPLC-MS/MS equipped with 2 LC-30AD pumps, a SIL-30AC autosampler, a CBM-20A communications bus module, a DGU-20A5R degassing unit, a CTO-30A column oven, and an 8050 triple-quadrupole mass spectrometer operated with a DUIS source. The separation was conducted with a Kinetex C8 column (150 x 3 mm, 2.6 μm; Phenomenex, Torrance, CA) under isocratic conditions with a mobile phase combination of 5% A (0.1% formic acid in water) and 95% B (0.1% formic acid in methanol). A flow rate of 0.5 mL/minute was used with a sample injection volume of 20 μL. Ionization occurred in the DUIS source with the following conditions: nebulizing gas flow of 2 L/min, heating gas flow of 10 L/min, interface temperature of 330C, DL temperature of 250C, heat block temperature of 400C, and a drying gas flow of 10 L/min. Cholesterol and [^13^C_2_]-cholesterol were analyzed with two m/z transitions each, 369.3/161.2 and 369.3/95.1 for cholesterol and 371.2/163.2 and 371.2/177.1 for [^13^C_2_]-cholesterol.

### Cholesterol efflux

SCC-61 and rSCC-61 cells were plated 2.0 x 10^5^ cells in 0.5 mL complete media (DMEM/F12, 10% FBS, 0.5% PenStrep) and allowed to adhere before labeling with 2 µCi/mL [^3^H]cholesterol (PerkinElmer) in DMEM/F12 containing 0.5% PenStrep and 2.5% FBS for 24 h. Cells were washed with 0.5 mL 1X DMEM (Gibco) containing 14 mM HEPES followed by 20 h equilibration in DMEM/F12 with or without 0.30 mM adenosine 3’,5’-cyclic monophosphate (cAMP). Cells were washed again with 0.5 mL 1X DMEM containing 14 mM HEPES and subsequently incubated with 0.5 mL MEM-HEPES (Gibco) supplemented with or without 0.15 mM cAMP and 30 μg/mL apolipoprotein A1 (APOA1) for 4 h. Following 4 h incubation, 0.5 mL of media was removed and 0.1 mL of media combined with 5 mL Bio-Safe II scintillation fluid (Research Products Int.) to be counted. Each well was then washed 2X with 0.5 mL cold saline buffer followed by extracting lipids with 1 mL isopropanol overnight at RT on rotator. The lipid extract was dried under N_2_ and resuspended in 0.5 mL isopropanol. Then 0.1 mL of lipid extract was combined with 5 mL Bio-Safe II scintillation fluid (Research Products Int.) to be counted. ^3^H-radioactivity was counted in medium and cellular lipid extract and cholesterol efflux was calculated as the percentage of ^3^H-radioactivity in medium/(^3^H-radioactivity in medium + ^3^H-radioactivity in the cellular lipid extract) * 100%.

### Immunostaining of lipid rafts and measurement of intracellular cortisol

2x10^4^ cells/well of shMTHFD2 knockdown radiation resistant rSCC-61 cells and shRNA control rSCC-61 cells were plated in Microtek chambered slide and were cultured in DMEM/F12 media containing 10% FBS and 1% penicillin/streptomycin at 37°C using a 5% CO_2_ incubator overnight. Next day, cells were treated with 10 μM Methotrexate for 2 hr at 37°C. After completion of incubation period, cells were washed with ice cold medium and incubated with 1 μg/mL of AlexaFluor594 conjugated CT-B for 15 min on ice. Further, the cells were washed twice with ice cold Dulbecco’s phosphate-buffered saline (DPBS) and fixed with 4% formaldehyde for 20 min and permeablized in 0.1% Triton X-100 for 10 min, washed, and mounted using Fluoromount (Sigma) for imaging. Imaging was performed using a Zeiss LSM710 confocal microscope and a 40X objective. The images were processed using the LSM image browser. Total cortisol was determined in SCC-61, rSCC-61 and MTHFD2 KD rSCC-61 cells by using 96 well plate ELISA kit, commercially available in Cayman Chemicals, USA (catalogue number. 500360).

### Statistical analysis

Data are presented as mean ± SEM. All statistical analyses were performed using SigmaPlot v.12. All data are from three independent experiments unless stated otherwise. Statistical significance of differences was evaluated with the student’s t test unless stated otherwise. Significance was accepted at the level of p < 0.05.

## Data availability statement

The data presented in the study are deposited in the ProteomeXchange PRIDE repository, accession number PXD029053. Source codes for the COSMro and FBA analysis are available at GitHub: https://github.com/JakeJiUThealth/MIP_V1.0 and https://github.com/kemplab/COSMro-FBA-modeling.

## Author contributions

Conceptualization CF and AT. Methodology CF, XZ, ZJ, JP, RB, MK. Investigation JM, ND-B, HW, KS, EL, VV, JL, C-CK. Writing-original manuscript JM, ZJ, CF. Writing – editing, all authors. Supervision CF, XZ, MK, RB, JP. Resources RB, JP. Funding, MK, CF, XZ. All authors contributed to the article and approved the submitted version.
