# Supplementary material for: Redox integration of signaling and metabolism in a head and neck cancer model of radiation resistance using COSMRO
Source: Front Oncol. 2023 Jan 4;12:946320. doi: 10.3389/fonc.2022.946320 (PMC9846845; doi:10.3389/fonc.2022.946320)
Supplement: Supplementary file 1 [file DataSheet_1.docx]

**Supplementary Material**

**Redox Integration of Signaling and Metabolism in a Head and Neck Cancer Cell Model of Radiation Resistance using COSM^RO^**

Zhiwei Ji^1†^, Jade Moore^2†^, Nelmi O. Devarie-Baez^2^, Joshua Lewis^3,4^, Hanzhi Wu^2^, Kirtikar Shukla^2^, Elsa I. Silva Lopez^2^, Victor Vitvitsky^5^, Chia-Chi Chuang Key^2^, Mercedes Porosnicu^6^, Melissa L. Kemp^3,4^, Ruma Banerjee^5^, John S. Parks^2^, Allen W. Tsang^2^, Xiaobo Zhou^1*^, and Cristina M. Furdui^2*^

^1^Division of Radiologic Sciences – Center for Bioinformatics and Systems Biology, Wake Forest University School of Medicine, Winston-Salem, NC, United States, ^2^Department of Internal Medicine, Section on Molecular Medicine, Wake Forest University School of Medicine, Winston-Salem, NC, United States, ^3^The Parker H. Petit Institute of Bioengineering and Bioscience, Georgia Institute of Technology, Atlanta, GA, United States, ^4^The Wallace H. Coulter Department of Biomedical Engineering, Georgia Institute of Technology, Emory School of Medicine, Atlanta, GA, United States, ^5^Department of Biological Chemistry, University of Michigan Medical School, Ann Arbor, MI, United States, ^6^Department of Internal Medicine, Section on Hematology and Oncology, Wake Forest University School of Medicine, Winston-Salem, NC, United States

^#^ Equal contribution authors

^*^ Co-corresponding authors

Contact: Cristina M. Furdui; Email: [cfurdui@wakehealth.edu](mailto:cfurdui@wakehealth.edu)

COMPUTATIONAL PROCEDURES

**Construction of a generic redox-regulated pathway map for SCC-61 and rSCC-61 cells.** The information obtained from four datasets (proteomics (Bansal et al., 2014), metabolomics, redox proteomics and protein phosphorylation described above and summarized in **Supplementary Dataset S1-S3** and **Figures S1-S3**) was used to build a redox-regulated generic network of integrated signaling and metabolic subnetworks (**Figure 1** and **Figure S4**). For the signaling subnetwork, we first selected the enriched signaling pathways generated from proteomics data. Other signaling pathways associated with the HNSCC radiation resistance phenotype were manually added such as the epidermal growth factor receptor (EGFR) pathway (De Carvalho et al., 2013; Psyrri et al., 2013), insulin-like growth factor 1 receptor (IGF1R) pathway (Papaconstantinou, 2009), tumor necrosis factor receptor (TNFR) pathway (Toyozumi et al., 2004), c-Jun N-terminal kinases (JNK)/p53 pathway (De Carvalho et al., 2013; Toyozumi et al., 2004), and nuclear factor kappa-light-chain-enhancer of activated B cells (NF-κB) pathway (Farshadpour et al., 2012). The metabolic subnetwork was built using the combined proteomics and metabolomics data, and Ingenuity Pathway Analysis (IPA, Qiagen). The Biochemical Genetic and Genomic (BiGG) database was used to annotate each metabolic reaction as reversible or irreversible (Schellenberger et al., 2010). Lastly, the network was annotated with proteins connecting signaling and metabolic subnetworks (blue filled circles, **Figure S4**), which included superoxide dismutase 2 (SOD2), phosphoglucomutase-1 and -2, cytidine-5’-triphosphate synthase (CTPS), thymidine kinase 1 (TK1), glucose-6-phosphate dehydrogenase (G6PD), pyruvate kinase isozyme M2 (PKM2), 3-hydroxy-3-methylglutaryl-coenzyme A reductase (HMGCR), squalene synthase (SQS/FDFT1), phosphoglucomutase (PGM) 1/2 and serine hydroxymethyltransferase 2 (SHMT2). The effects of oxidation and phosphorylation on the activity of the proteins in the resulting network were annotated manually based on literature data. Seven of the proteins were identified as being under redox-regulation in SCC-61 and rSCC-61 cells based on the redox proteomics data: signal transducer and activator of transcription 3 (STAT3), heat shock protein 90 (HSP90), eukaryotic elongation factor 2 (eEF2), NF-κB, glutamate-cysteine ligase catalytic subunit (GCL), EGFR, and PKM2 (**Supplementary Table S1**). We also considered the redox-sensitive target Akt2 (Wani et al., 2011). The eight redox-regulated signaling proteins and metabolic enzymes were connected to a node “H_2_O_2_” in the network according to the information in **Supplementary Table S2**.

Next, we represented signaling as a Boolean network consisting of a set of nodes and a set of directed edges (Ji et al., 2014; Mitsos et al., 2009). Nodes in the signaling subnetwork represent signaling proteins (including some metabolic enzymes with dual function in signaling) and the edges represent activation (->) or inhibition (-|) effects on downstream proteins. Examples of multiple activation, inhibition or mixed effects on downstream proteins are illustrated in **Figure S5A**-**D**. Similar to the signaling subnetwork, the metabolic subnetwork topology consists of a set of nodes and a set of edges. In this case, the nodes denote the metabolites in biochemical reactions and their measurements as ratios of concentrations in rSCC-61 relative to SCC-61. The edges indicate the metabolic reactions that are controlled by corresponding metabolic enzymes. The edges with duplex arrows denote reversible metabolic reactions, while unidirectional arrows denote irreversible reactions. The list of all metabolic subnetwork components including thermodynamic parameters for each metabolic reaction extracted from NIST Standard Reference Database (Goldberg et al., 2004), BiGG (Schellenberger et al., 2010), and Kyoto Encyclopedia of Genes and Genomes (KEGG) (Kotera et al., 2012) is included in **Supplementary Dataset S4**.

The resulting topology of the generic integral network is shown in **Figure S4**. The upper portion of **Figure S4** is the signaling subnetwork, which consists of 54 signaling proteins (22 measured) and 10 metabolic enzymes (5 measured) connected through 87 regulatory signaling events (**Supplementary Dataset S5**). The lower portion of **Figure S4** is the metabolic sub-network which contains 74 metabolic reactions (37 metabolic enzymes measured) and 107 metabolites (38 metabolites measured).

**Mixed Integer Programming (MIP).** In the integral network topology, the signaling subnetwork is defined as a set of signaling proteins$=\{1,2,\ldots,j,\ldots n_{s}\}$ and reactions$=\{1,2,\ldots,i,\ldots n_{r}\}$. The signaling proteins are receptors, kinases, phosphatases and transcription factors. In our study, a subset ($SE$) of enzymes bridging the signaling and metabolic subnetworks were also considered as components of the signaling subnetwork. The $i$ –th signaling reaction is defined as $j\to p$ ($j,p\in$), which connects two proteins: the upstream protein$j$, and the downstream protein $p$. If several signaling reactions connect with the same protein $p$, the relationship between these reactions were logic “OR” which indicate that the state ( $x_{p}$ ) of protein $p$ might be regulated by at least one of its upstream proteins.

In our MIP approach, a binary variable $x_{j}\in\{0,1\}$ indicates if the protein $j$ is up-regulated ($x_{j}=1$) or down-regulated ($x_{j}=0$) in rSCC-61 compared with SCC-61. The variable $z_{i}$ denotes if the reaction $i$ takes place ($z_{i}=1$) or not ($z_{i}=0$). In the signaling pathway topology, there are two types of reactions: activation and inhibition. When activation takes place, which means the changes in the activity of protein $p$ are positively related to that of its upstream protein $j$, the state of $z_{i}$ can be represented with a logic operation (the reverse “exclusive OR”) between$x_{ij}$ and $x_{ip}$ (Eq. (2)). Similarly, the increased upstream protein $x_{ij}$ might lead to down-regulation of downstream protein $x_{ip}$ via inhibition. Thus, the state of $z_{i}$ equals logic “exclusive OR” between $x_{ij}$ and $x_{ip}$ (Eq. (3)).

The metabolic sub-network is defined as a set of metabolites$=\left\{ 1,2,\ldots,k,\ldots m_{s} \right\}$and a set of metabolic reactions$=\{1,2,\ldots,l,\ldots m_{r}\}$. The concentration of metabolite $k$ is represented with a continuous variable $C_{k}$, in which $k\in$. Each metabolic reaction $l$ has two corresponding index sets, substrates $S_{l}$ and products $P_{l}$, which are subsets of $M$ ($S_{l} ,P_{l} \subset M$). In our study, an enzyme was associated with one metabolic reaction; hence, we applied the enzyme states to represent the states of corresponding reactions in our model. The set ($E$) of binary variables is defined to denote all the metabolic enzymes in our network, where$=\{e_{1},e_{2},\ldots,e_{l},\ldots,e_{m_{r}}\}$. A subset ($SE$) of set $E$ denotes the enzymes connecting signaling and metabolic subnetworks. Thus, the enzymes in $SE$ belong to both subnetworks (). With regard to the metabolic reaction $l\in$ , the binary variable $e_{l}\in\{0,1\}$ indicates if the state of $l$-th enzyme is up-regulated ($e_{l}=1$) or down-regulated ($e_{l}=0$) in rSCC-61 cells. In our model, down-regulated enzymes potentially block the corresponding metabolic reactions. The flux value and the change in Gibb’s free energy of reaction $l$are denoted with continuous variables $v_{l}$, and${\Delta G}_{l}$, respectively. We first performed flux balance analysis (FBA) to ensure the mass or energy balance in the whole network (Orth et al., 2010; Tepper et al., 2013). Thermodynamic constraints were then applied to restrict the consensus rule that the net flux of a chemical reaction and the change of Gibb’s free energy are related to each other: $sgn\left( v \right)=-sgn(\Delta G)$ (Hoppe et al., 2007).

For inferring the rSCC-61 specific network, we used our COSM^ro^ approach to find an optimal solution for the integral pathway network. We considered two types of elements in our optimization: The first was to minimize the difference between predicted values and measurements of species, which includes signaling proteins, metabolic concentrations, and the states of enzymes. The second objective is to obtain a maximal sub-graph of the generic network. The objective function is defined as:


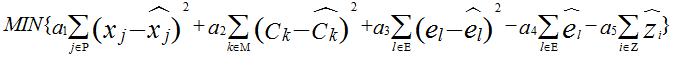
 (1)

where $\hat{(\bullet)}$ and are the predicted and measured values, respectively. The positive parameters ($a_{1}$,$a_{2}$, $a_{3}$,$a_{4}$,$a_{5}$) in Eq. (1) control the weight of corresponding objectives. Therefore, the first three parameters ($a_{1}$,$a_{2}$, $a_{3}$) minimized the differences between measurements and predicted values, including the states of signaling proteins, concentrations of metabolites, and the states of enzymes in the whole network. The last two parameters ($a_{4}$ and$a_{5}$) optimized the scale of inferred network topology aiding in removal of inconsistent reactions. The constraints in our model can be summarized as:

| $z_{i}=\overline{x_{ip}\oplus x_{ij}}, i\in\{1,\ldots,n_{r}\}, j,p\in\{1,2,\ldots,j,\ldots n_{s}\}$ | (2) |
| --- | --- |
| $z_{i}=x_{ip}\oplus x_{ij}, i\in\{1,\ldots,n_{r}\}, j,p\in\{1,2,\ldots,j,\ldots n_{s}\}$ | (3) |
| $z_{i}\geq x_{ij}, i=1,\ldots,n_{r}, j\in\{1,2,\ldots,j,\ldots n_{s}\}$ | (4) |
| $S*V=0$ | (5) |
| $\frac{{\Delta G}_{l}}{RT}=\left( \sum_{k1\in P_{l}} \ln(C_{k1})-\sum_{k2\in S_{l}} \ln(C_{k2}) \right)-ln[K_{eq}^{l}]$ | (6) |
| $\left\{ \begin{aligned} C_{min}\leq C_{k1}\leq C_{max}, k1\in P_{l} \\ C_{min}\leq C_{k2}\leq C_{max}, k2\in S_{l} \end{aligned} \right.$ | (7) |
| $0\leq v_{l}\leq e_{l}v_{max}$, $l\in$ | (8) |
| $\Delta G_{l}\leq\varepsilon\left( 1-e_{l} \right)$ | (9) |
| $\left\{ \begin{aligned} C_{kt}+\Delta\delta\left( e_{l}-1 \right)\geq M_{kt}, kt\in P_{l} \\ C_{kd}+\Delta\delta\left( 1-e_{l} \right)\leq M_{kd}, kt\to\to kd \end{aligned} \right.$ | (10) |
| $v_{min}\leq v_{l}\leq v_{max}$ | (11) |
| $-v_{l}+d^{+}\left( \varepsilon-v_{min} \right)\leq-v_{min}$ | (12) |
| $\Delta G_{l}+d^{+}\left( \varepsilon-v_{min} \right)\leq-v_{min}$ | (13) |
| $v_{l}+d^{-}(\varepsilon+v_{max})\leq v_{max}$ | (14) |
| $-\Delta G_{l}+d^{-}(\varepsilon+v_{max})\leq v_{max}$ | (15) |
| $e_{l}=d_{l}^{+}+d_{l}^{-}$ | (16) |
| $X\in\left\{ 0,1 \right\}^{n_{s}},Z\in\left\{ 0,1 \right\}^{n_{r}},{E\in\left\{ 0,1 \right\}^{m_{r}}, d}_{l}^{+},d_{l}^{-}\in\{0,1\}$ | (17) |

According to these formulas, the constraints (2-3) are used to infer the states of all the signaling reactions (Eq. (2) activation, Eq. (3) inhibition). The constraint (4) was used to restrict the states of a reaction when it uniquely promotes a terminal protein. The system of mass or energy balance equations at steady state is given in constraint (5). The metabolic reactions are represented as a stoichiometric matrix ($S$) and the flux vector was represented as$V$. The constraint (6) indicates the changes in Gibb’s free energy calculated from changes in standard Gibb’s energy, where $C_{k1}$ and $C_{k2}$ are the active concentrations (activity) of the metabolites in reaction $l$. $S_{l}$ and $P_{l}$ denote the sets of substrates and products of the reaction $l$, respectively. *R* is universal gas constant and *T* is the absolute temperature. The values for all parameters were extracted from NIST Standard Reference Database (Goldberg et al., 2004), HMDB (Wishart et al., 2007) and Uniprot (Dimmer et al., 2012; O'Donovan and Apweiler, 2011). Constraint (7) is used to restrict the concentration values of all metabolites in the network. Thermodynamic constraint (8) ensures that the net flux $v_{l}$ is restricted between $0$ and $v_{max}$ as lower and upper bounds if the irreversible reaction $l$ is activated (the state of enzyme $x_{E}$ is up-regulated); otherwise, the flux value is zero. Equations (8)-(9) ensure the opposite relationship between direction of the net flux and the change of Gibb’s free energy when the reaction is un-blocked in an irreversible metabolic reaction. Moreover, if the enzyme in reaction $l$ was directly blocked by an inhibitor, the substrates ($C_{kt}$) of reaction $l$ would be accumulated and the expression of all the metabolites ($C_{kd}$) in downstream of reaction $l$ would be significantly down-regulated. Constraint (11) is also used to restrict the flux values in the reversible reactions. Similarity, constraints (12) -(15) ensure the direction of net flux and Gibb’s energy in reversible reactions. The constraint (16) restricts the flux of a reversible reaction to one direction if the corresponding enzyme is up-regulated. Because variables $e_{l}$, $d_{l}^{+}$ and $d_{l}^{-}$ are all binary, it is possible to infer the unique direction of flux in a reversible reaction. The formula (17) restricts the values of three groups of binary variables$X$, $Z$ and $d_{l}^{+}$, $d_{l}^{-}$. Since our data reflects the changes in the activities of proteins between two cell lines, there is only one data point in the analysis. Different from the ODE-based approaches (Lu et al., 2011), our model described here can quickly infer a large-scale cell-specific network by fitting the measurements to a network topology.

**Determine the changes of protein activities combining phosphorylation, oxidation, and total expression.** For our network, we estimated the qualitative changes of activities for seven signaling proteins, in which four proteins were screened from the redox proteomics data (**Supplementary Dataset 3**) and two screened from the literature. Detailed information is presented in **Supplementary Table S3**.

1. **EGFR**. It is known that EGFR can be activated by both oxidation and phosphorylation. In our experimental data, the rSCC-61/SCC-61 phosphorylated EGFR (pEGFR) ratio was 0.4, indicating lower EGFR activity in rSCC-61. Considering rSCC-61 has a lower concentration of reactive oxygen species (ROS) than in SCC-61, it follows that the oxidation state of EGFR in rSCC-61 should also be lower than in SCC-61. The rSCC-61/SCC-61 BP1-labeled EGFR ratio of 0.637 determined by the redox proteomics studies confirmed this result. Therefore, lower oxidation in rSCC-61 also contributes to lower EGFR activity in these cells. Combining the effects of phosphorylation and oxidation, we inferred EGFR activity is down-regulated in rSCC-61 relative to SCC-61 cells.
2. **STAT3**. STAT3 is inactivated by oxidation and activated by phosphorylation. Phosphorylation data showed increased phosphorylation of STAT3 in rSCC-61 cells compared to SCC-61 cells. However, the oxidation ratio of STAT3 is 0.5534. Therefore, we inferred the ratio of STAT3 activity is upregulated due to decreased oxidation but increased phosphorylation in rSCC-61 cells compared to SCC-61 cells and increased phosphorylation status.
3. **eEF2**. eEF2 is inactivated by oxidation and activated by phosphorylation. The phosphorylation ratio is 1.99 while the oxidation ratio is 0.803. Therefore, we inferred the ratio of EEF2 is up-regulated.
4. **HSP90**. ROS also can inhibit HSP90. Only the measurements of total expression and oxidation were available. Oxidation ratio is decreased (0.804), and total expression of this protein is significantly increased (1.798). Therefore, HSP90 was considered to be up-regulated in rSCC-61.
5. **AKT2**. Akt2 is inhibited by the accumulated ROS and is phosphorylated by PI3K/PDK pathway. The ratio of pAKT2 is 0.82. We used our proposed model to determine the activity of Akt2.
6. **SHMT2**. The total expression of ROS is sharply increased (4.0125) potentially due to lower ROS in rSCC-61. Thus, we consider oxidation inhibits SHMT2 activity.
7. **PKM**. Similar as SHMT2, we also inferred that PKM activity is up-regulated in rSCC-61 because the increased total expression (1.165) induced by ROS.

**Flux balance analysis.** Parsimonious flux balance analysis was performed using the COBRA software package (Ebrahim et al., 2013). We sought to maximize the conversion from cytoplasmic NADP^+^ to cytoplasmic NADPH, and see which NADPH-producing reactions in the solution carried the largest flux. This is equivalent to asking: if the cell is converting as much NADP^+^ to NADPH as possible, through which pathways will this occur? This objective function is implemented by adding the reaction *x*: nadph_c → nadp_c to the metabolic model, and then maximizing the flux through this reaction with the constraints of the following equations

max *v_x_*_,_ where *x*: nadph_c →nadp_c

subject to *S*$*$**v** = **0** (5)

and lb*_i_* ≤ *v_i_* ≤ ub*_i_*

where S is the stoichiometric matrix of *m* metabolites and *r* reactions, **v** is a *r* x 1 vector of reaction fluxes, and *lb_i_* and *ub_i_* indicate the lower bound and upper bound of each given flux *v_i_*. The objective function will cause other reactions in the model that produce cytoplasmic NADPH from NADP^+^ to have very high flux (to balance out the consumption of cytoplasmic NADPH and production of NADP^+^ in the maximized reaction *x*).

*Media constraints*. In HMR 2.0 (Mardinoglu et al., 2014), exchange reactions exist between the different compartments of the model. Exchange reactions between C_x and C_e, and then between C_e and C_c, provide the cell with nutrients and metabolic precursors from its environment. The media within which the cell lives is often modeled as the upper bounds of metabolite exchange from C_x to C_e. For example, to model a low-glucose environment, a small upper bound for glucose exchange between C_x and C_e is set. Traditionally in FBA, media constraints imposed on the cell are limited to setting a small upper bound for a few “limiting metabolites” (usually including glucose), and making all other metabolites freely-available (by setting the upper bound to the maximum possible flux value). However, a more accurate representation of the media constraints on cellular metabolism can be modeled by setting upper bounds for all available metabolites in proportion to their concentration in the media. We modeled ThermoFisher’s DMEM/F-12 media by setting the upper bound for each metabolite exchange reaction from C_x to C_e in proportion to its concentration in the actual media; for C_x to C_e exchange reactions involving metabolites not in the media, we set the upper bound to zero. Each upper bound (in units of mmol/gDW/hr) is set equal to the numerical value of the metabolite’s media concentration in mmol/L.

*Context-specific models (HNSCC).* Human Metabolic Reaction (HMR) 2.0 model (Mardinoglu et al., 2014) contains 5546 metabolites, 8181 reactions, and 3765 reaction-associated genes found within all cells of the human body; however, in order to study the redox metabolism of a particular cell type or cancer type, we must create context-specific models of these different cells. This can be accomplished by removing reactions in HMR 2.0 that do not normally occur within the particular cell type. To create context-specific models of HNSCC cancer, proteomic data from the Human Protein Atlas (HPA) was used (Uhlen et al., 2015). The expression status of a gene was set to “Expressed” if at least 50% of the biopsy results had a HPA expression level of High, Medium, or Low. If at least 50% of the biopsy results had a HPA expression level of Not Detected, the expression status of the gene was set to “Not Expressed”. These gene expression statuses were used to determine which reactions from the original HMR 2.0 model would be removed or not removed in the context-specific model. SCC-61/rSCC-61 specific models were generated by Illumina transcriptomics and compared to a catalog of 196 HPV-negative HNSCC samples (Wichmann et al., 2015) with the resulting cell line models containing 6,789 (SCC-61) and 6,596 (rSCC-61) reactions. Optimization was performed independently for maximal cytosolic and mitochondrial NAD(P)H production.

Each reaction in HMR 2.0 has an associated gene reaction rule, written as a Boolean function of the genes whose products execute the reaction (for example, the gene reaction rule can be a Boolean function of the genes representing different isoforms of the enzyme which catalyzes the reaction). To determine whether a particular reaction should be removed from HMR 2.0 to build the context-specific model, the expression status of each gene was placed in the gene reaction rules (Expressed = True, Not Expressed = False). If the resulting Boolean function came out to be True, then the genes necessary to execute the reaction are being expressed in the tissue, and the reaction is kept in the context-specific model. If the Boolean function came out to be False, then the necessary genes are not expressed, and the reaction was removed from the context-specific model.

*Context-specific models (SCC-61, rSCC-61).* Triplicate transcriptomic data from rSCC-61 and SCC-61 cells were log2 transformed and normalized by robust spline normalization. We aggregated HNSCC transcriptomic data into a single Illumina dataset by performing batch correction between our data and the HPV- samples from (Wichmann, 2015) using ComBat (Kitchen 2010). Using the HNSCC model as the starting point for our cell line-specific models, we performed the following steps:

1. If the reaction is not associated with any gene, keep the reaction ON;
2. If the reaction is associated with a gene(s), but there are no Illumina probes in the aggregated data set associated with the gene(s), use the same reaction ON/OFF status as in the original head-neck cancer model;
3. If the reaction is associated with a gene(s), and there are Illumina probe(s) in the data sets associated with the gene(s): initialize a score for the gene for both SCC-61 and rSCC-61

For each gene:

a) Initialize a score for the gene for both SCC-61 and rSCC-61: score_SCC = 0, score_rSCC = 0

b) For each Illumina probe associated with the gene:

If ALL 3 of the SCC-61 values are > 1 standard deviation (of the 196 Wichmann samples) from the mean (of the 196 Wichmann samples), ADD 1 point to the gene's SCC-61 score.

If ALL 3 of the SCC-61 values are < 1 standard deviation from the mean, SUBTRACT 1 point to the gene's SCC-61 score. Otherwise, don't change the gene's SCC-61 score.

Repeat for the rSCC-61 values and gene's rSCC-61 score

c) If the gene's SCC-61 score is > 0, make the gene's expression status ON.

d) If the gene's SCC-61 score is < 0, make the gene's expression status OFF. Otherwise, use the same expression status as the original head-neck cancer model.

d) Repeat for rSCC-61

4) Use the reaction's Boolean gene reaction rule (eg. gene 1 AND gene 2) to set the reaction ON/OFF status in the SCC-61 and rSCC-61 models.

SUPPLEMENTARY DATASETS

**Dataset S1.** Network metabolites including the 38 metabolites quantified in the SCC-61 and rSCC-61 cells.

**Dataset S2.** Differentially sulfenylated redox proteins in SCC-61 and rSCC-61 cells.

**Dataset S3.** Summary of measured ratios of total protein expression and protein phosphorylation in rSCC-61 and SCC-61 cell along with the known consequence on protein activity.

**Dataset S4.** Summary of thermodynamic parameters for each metabolic reaction extracted from NIST Standard Reference Database, BiGG, and KEGG.

**Dataset S5.** List of reactions in the signaling subnetwork, which consists of 54 signaling proteins (22 measured) and 10 metabolic enzymes (5 measured) connected through 87 regulatory signaling events.

SUPPLEMENTARY TABLES

**Table S1.** Subset of network proteins identified in the redox proteomics data.

**Table S2.** Redox-regulated signaling and metabolic proteins connected to node “H_2_O_2_” and the effects of oxidation on their activity.

**Table S3.** Differential expression, phosphorylation, oxidation and predicted activity changes of validated proteins.

**Table S4.** Primers and PCR conditions for quantification of β-catenin.

SUPPLEMENTARY FIGURES

**Figure S1.** **Metabolomics Analysis.** **A.** Principal component analysis plot of differential metabolite data that characterizes the trends exhibited by SCC-61 (black) and rSCC-61 (red). Each dot represents a sample. **B.** Supervised partial least square discriminant analysis. **C.** Selected list of top 25 differential metabolites in rSCC-61 versus SCC-61 cells.


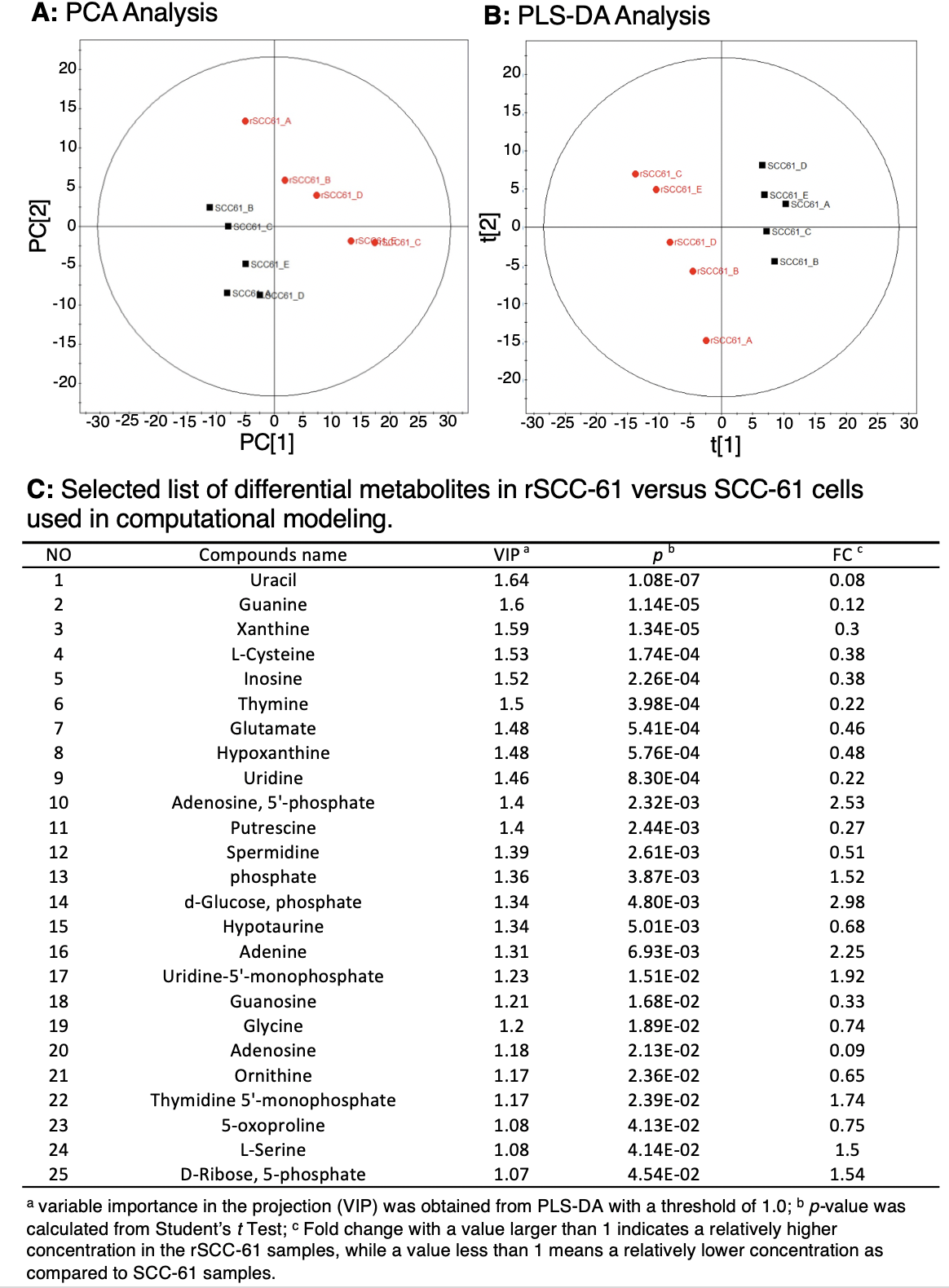


**Figure S2.** **Redox Proteomics. A.** Chemical structure of biotin-tagged 1,3-cyclopentanedione (BP1) probe. **B.** nanoLC-MS and MS/MS workflow to label sulfenylated proteins (-SOH) with BP1 and quantify by MS. **C.** Plot of log2 normalized protein ratio showing differential protein sulfenylation in rSCC-61 vs SCC-61. **D.** Ingenuity Pathway Analysis (IPA) showing distribution of proteins subcellular locations (**upper**)**,** and distribution of molecular functions **(lower)** of sulfenylated proteins in rSCC-61 vs SCC-61 cells.


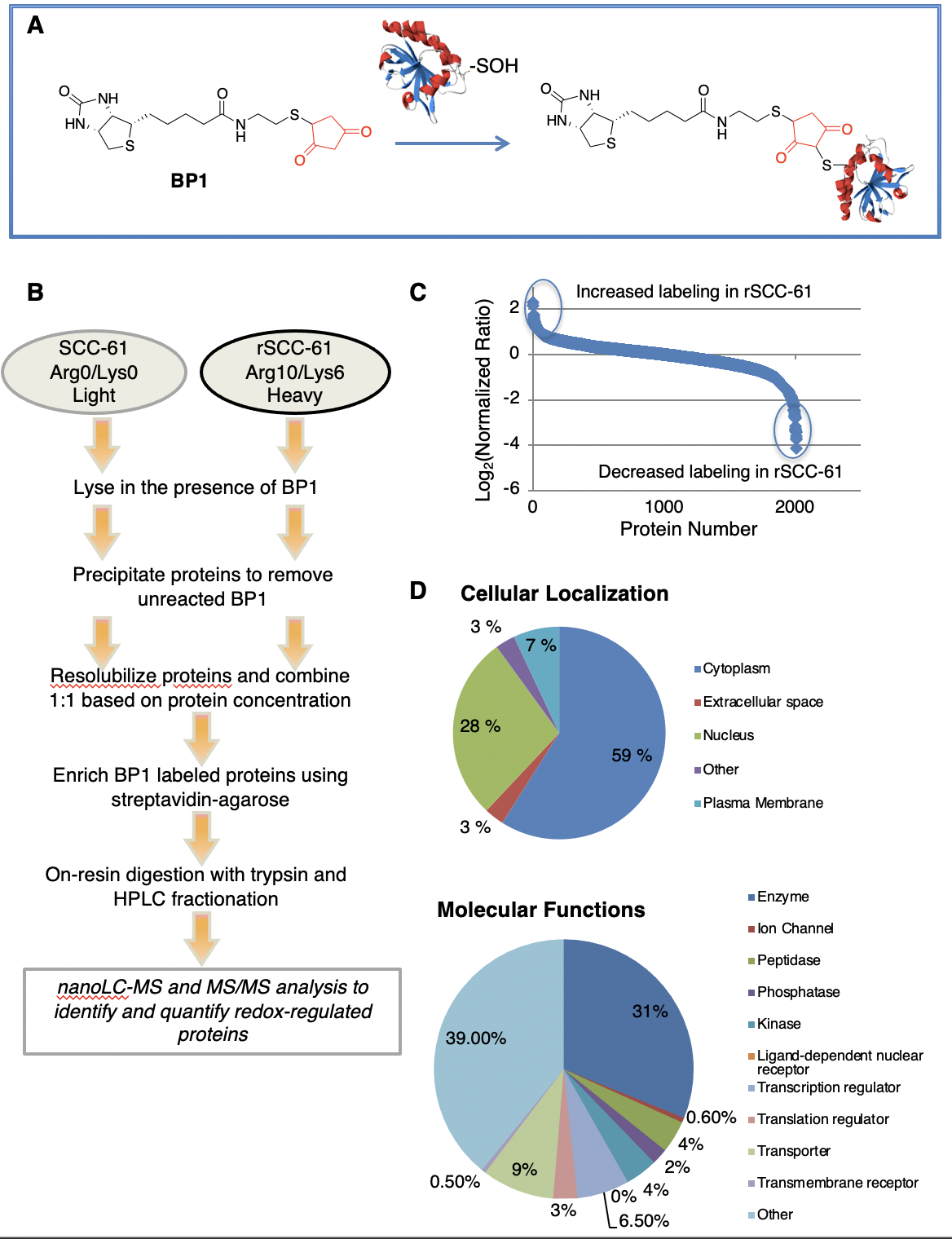


**Figure S3.** **Targeted Western Blot Analysis. A.** Western blot analysis to determine differential expression of TIGAR, TP53, PRX1, PRX-SO_2/3_, and SRX in rSCC-61 vs SCC-61 cells. **B.** Western blot analysis to determine differential phosphorylation of IGFR, JNK1, Myc, RPS6, PKC, Akt, PDK1, STAT3, ASK1, LKB1, STAT1, and 4E-BP1 in rSCC-61 vs SCC-61 cells. *p* values *0.01-0.05, **0.01-0.001, ***<0.001.

**
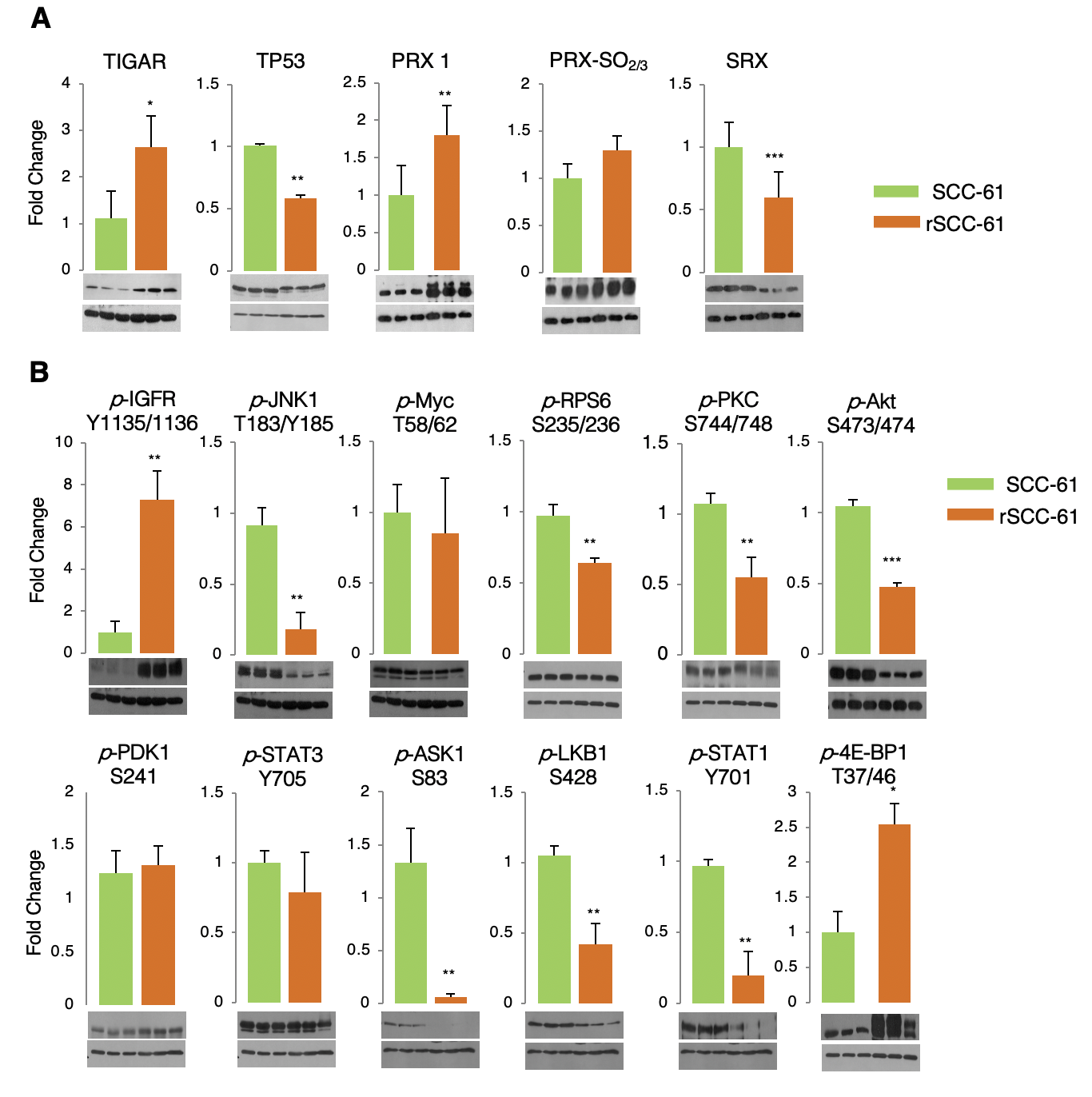
**

**Figure S4.** **Generic pathway Network.** Network composed of integrated signaling and metabolic networks by selecting enriched signaling pathways, metabolic pathways and redox-regulated pathways. Blue circles = enzymes connecting signaling and metabolic pathways. White box = signaling proteins. Orange line = metabolic reactions. Blue hashed line = enzyme regulation. Black hashed line = redox-regulated pathways. Blue line = signaling reactions.


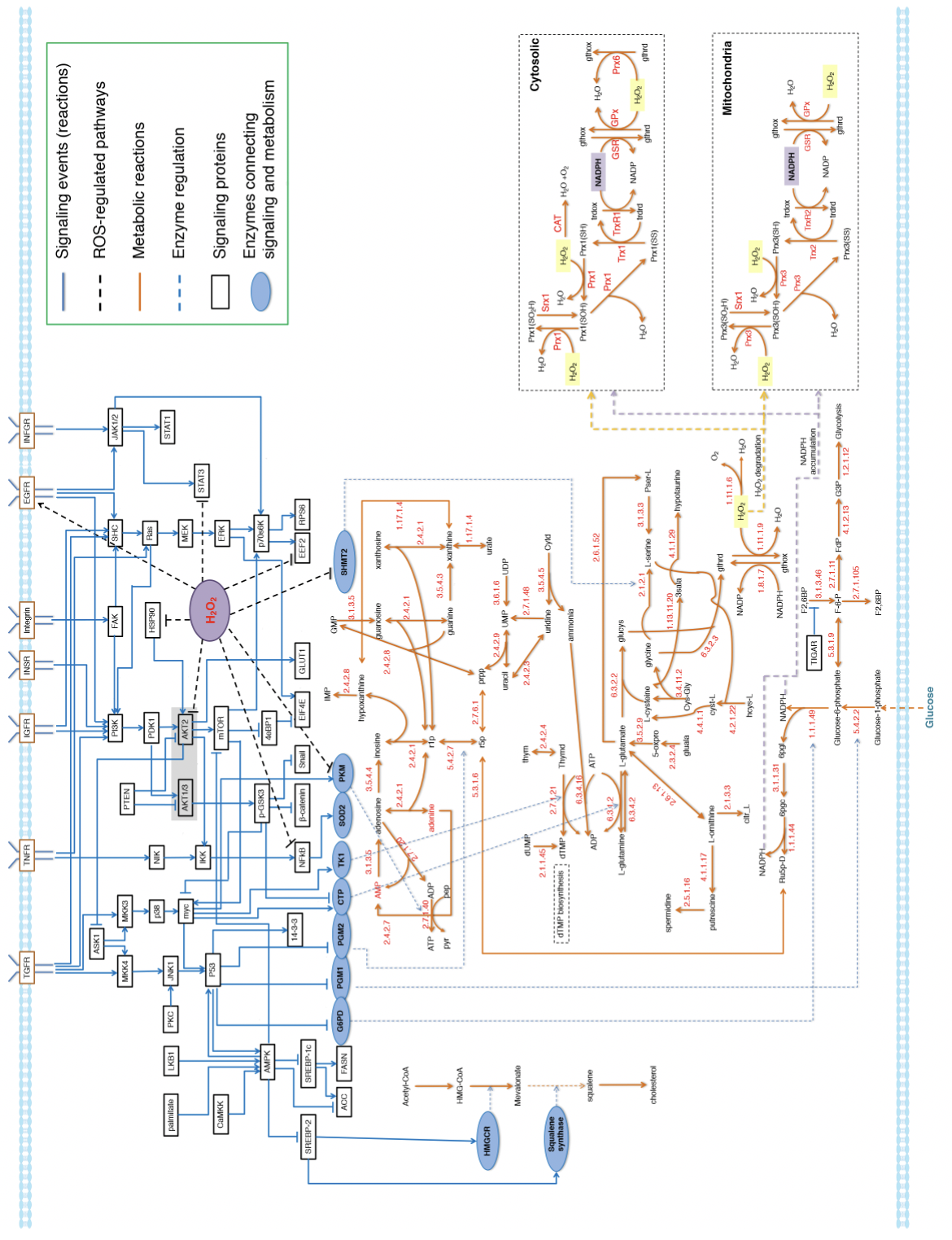


**Figure S5.** **Signaling Subnetwork Represented as a Boolean Network. A.** Schematic and example of multiple promotions. **B.** Schematic and example of multiple inhibitions. **C.** Schematic and example of mixed reactions. **D.** Schematic and example of metabolic enzyme inhibition.


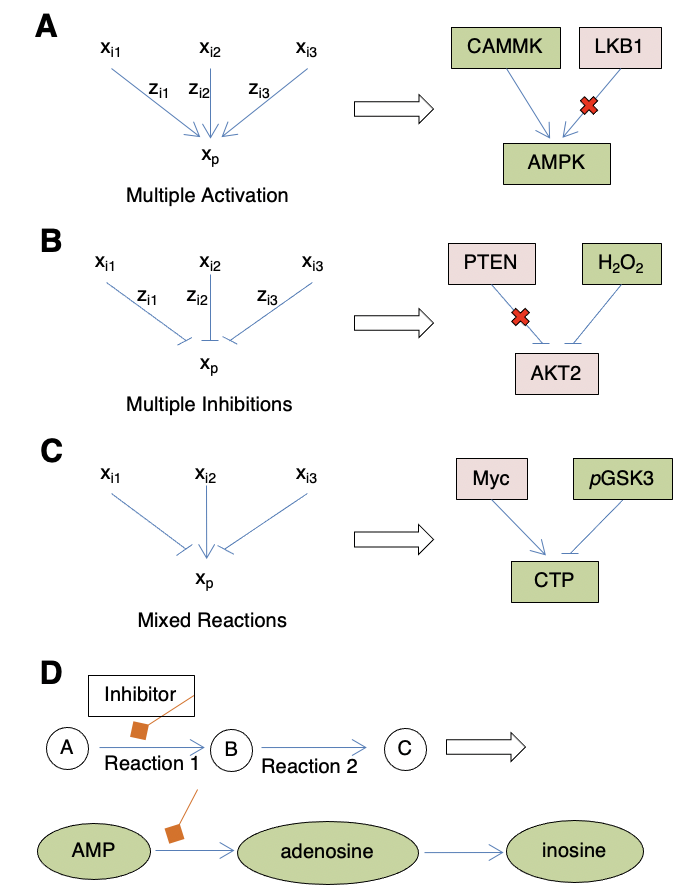


**Figure S6.** **Cholesterol Synthesis and Trafficking. A.** SREBP2 and LXR target genes extracted from the transcriptomics analysis of SCC-61 and rSCC-61 cells using the HumanHT-12 v4 Expression BeadChip. **B.** mRNA expression levels of genes that inactivate SREBP2 and oxysterols extracted from the same transcriptomics data as in (A). *p* values *0.01-0.05, **0.01-0.001, ***<0.001.


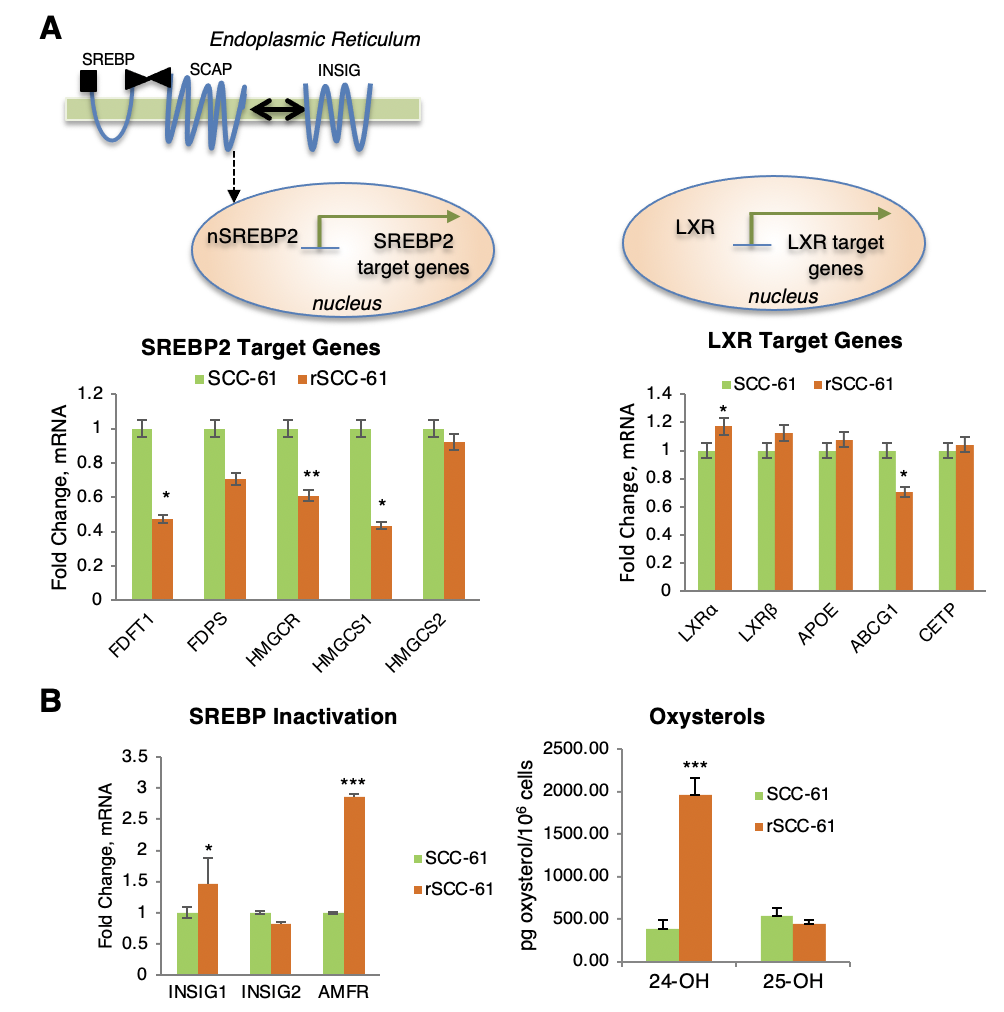


SUPPLEMENTARY REFERENCES

Bansal, N., Mims, J., Kuremsky, J.G., Olex, A.L., Zhao, W., Yin, L., Wani, R., Qian, J., Center, B., Marrs, G.S., et al. (2014). Broad Phenotypic Changes Associated with Gain of Radiation Resistance in HNSCC. Antioxid Redox Signal.

De Carvalho, T.G., De Carvalho, A.C., Maia, D.C., Ogawa, J.K., Carvalho, A.L., and Vettore, A.L. (2013). Search for mutations in signaling pathways in head and neck squamous cell carcinoma. Oncology reports *30*, 334-340.

Dimmer, E.C., Huntley, R.P., Alam-Faruque, Y., Sawford, T., O'Donovan, C., Martin, M.J., Bely, B., Browne, P., Mun Chan, W., Eberhardt, R., et al. (2012). The UniProt-GO Annotation database in 2011. Nucleic acids research *40*, D565-570.

Ebrahim, A., Lerman, J.A., Palsson, B.O., and Hyduke, D.R. (2013). COBRApy: COnstraints-Based Reconstruction and Analysis for Python. BMC Syst Biol *7*, 74.

Farshadpour, F., Roepman, P., Hordijk, G.J., Koole, R., and Slootweg, P.J. (2012). A gene expression profile for non-smoking and non-drinking patients with head and neck cancer. Oral diseases *18*, 178-183.

Goldberg, R.N., Tewari, Y.B., and Bhat, T.N. (2004). Thermodynamics of enzyme-catalyzed reactions - a database for quantitative biochemistry. Bioinformatics *20*, 2874-2877.

Hoppe, A., Hoffmann, S., and Holzhutter, H.G. (2007). Including metabolite concentrations into flux balance analysis: Thermodynamic realizability as a constraint on flux distributions in metabolic networks. Bmc Syst Biol *1*.

Ji, Z.W., Su, J., Liu, C.L., Wang, H.Y., Huang, D.S., and Zhou, X.B. (2014). Integrating Genomics and Proteomics Data to Predict Drug Effects Using Binary Linear Programming. Plos One *9*.

Kotera, M., Hirakawa, M., Tokimatsu, T., Goto, S., and Kanehisa, M. (2012). The KEGG databases and tools facilitating omics analysis: latest developments involving human diseases and pharmaceuticals. Methods in molecular biology *802*, 19-39.

Lu, T., Liang, H., Li, H., and Wu, H. (2011). High Dimensional ODEs Coupled with Mixed-Effects Modeling Techniques for Dynamic Gene Regulatory Network Identification. Journal of the American Statistical Association *106*, 1242-1258.

Mardinoglu, A., Agren, R., Kampf, C., Asplund, A., Uhlen, M., and Nielsen, J. (2014). Genome-scale metabolic modelling of hepatocytes reveals serine deficiency in patients with non-alcoholic fatty liver disease. Nat Commun *5*, 3083.

Mitsos, A., Melas, I.N., Siminelakis, P., Chairakaki, A.D., Saez-Rodriguez, J., and Alexopoulos, L.G. (2009). Identifying Drug Effects via Pathway Alterations using an Integer Linear Programming Optimization Formulation on Phosphoproteomic Data. Plos Comput Biol *5*.

O'Donovan, C., and Apweiler, R. (2011). A guide to UniProt for protein scientists. Methods in molecular biology *694*, 25-35.

Orth, J.D., Thiele, I., and Palsson, B.O. (2010). What is flux balance analysis? Nat Biotechnol *28*, 245-248.

Papaconstantinou, J. (2009). Insulin/IGF-1 and ROS signaling pathway cross-talk in aging and longevity determination. Mol Cell Endocrinol. *299*.

Psyrri, A., Seiwert, T.Y., and Jimeno, A. (2013). Molecular pathways in head and neck cancer: EGFR, PI3K, and more. American Society of Clinical Oncology educational book / ASCO. American Society of Clinical Oncology. Meeting, 246-255.

Schellenberger, J., Park, J.O., Conrad, T.M., and Palsson, B.O. (2010). BiGG: a Biochemical Genetic and Genomic knowledgebase of large scale metabolic reconstructions. BMC bioinformatics *11*, 213.

Tepper, N., Noor, E., Amador-Noguez, D., Haraldsdottir, H.S., Milo, R., Rabinowitz, J., Liebermeister, W., and Shlomi, T. (2013). Steady-State Metabolite Concentrations Reflect a Balance between Maximizing Enzyme Efficiency and Minimizing Total Metabolite Load. PloS one *8*.

Toyozumi, Y., Arima, N., Izumaru, S., Kato, S., Morimatsu, M., and Nakashima, T. (2004). Loss of caspase-8 activation pathway is a possible mechanism for CDDP resistance in human laryngeal squamous cell carcinoma, HEp-2 cells. International journal of oncology *25*, 721-728.

Uhlen, M., Fagerberg, L., Hallstrom, B.M., Lindskog, C., Oksvold, P., Mardinoglu, A., Sivertsson, A., Kampf, C., Sjostedt, E., Asplund, A., et al. (2015). Proteomics. Tissue-based map of the human proteome. Science *347*, 1260419.

Wani, R., Qian, J., Yin, L., Bechtold, E., King, S.B., Poole, L.B., Paek, E., Tsang, A.W., and Furdui, C.M. (2011). Isoform-specific regulation of Akt by PDGF-induced reactive oxygen species. Proceedings of the National Academy of Sciences of the United States of America *108*, 10550-10555.

Wichmann, G., Rosolowski, M., Krohn, K., Kreuz, M., Boehm, A., Reiche, A., Scharrer, U., Halama, D., Bertolini, J., Bauer, U., et al. (2015). The role of HPV RNA transcription, immune response-related gene expression and disruptive TP53 mutations in diagnostic and prognostic profiling of head and neck cancer. Int J Cancer *137*, 2846-2857.

Wishart, D.S., Tzur, D., Knox, C., Eisner, R., Guo, A.C., Young, N., Cheng, D., Jewell, K., Arndt, D., Sawhney, S., et al. (2007). HMDB: the Human Metabolome Database. Nucleic acids research *35*, D521-526.
